# Quantitative analysis of phosphoproteome in necroptosis reveals a role of TRIM28 phosphorylation in promoting necroptosis-induced cytokine production

**DOI:** 10.1038/s41419-021-04290-7

**Published:** 2021-10-23

**Authors:** Rui Zu, Zhen Yu, Jing Zhao, Xiaojuan Lu, Wei Liang, Le Sun, Chenfang Si, Kezhou Zhu, Tian Zhang, Ganquan Li, Mengmeng Zhang, Yaoyang Zhang, Nan Liu, Junying Yuan, Bing Shan

**Affiliations:** 1 Interdisciplinary Research Center on Biology and Chemistry, Shanghai Institute of Organic Chemistry, Chinese Academy of Sciences, 100 Haike Road, PuDong District, 201210 Shanghai, China; 2 University of Chinese Academy of Sciences, Beijing, China

**Keywords:** Cell biology, Immunology

## Abstract

Necroptosis is a form of regulated necrotic cell death that promotes inflammation. In cells undergoing necroptosis, activated RIPK1 kinase mediates the formation of RIPK1/RIPK3/MLKL complex to promote MLKL oligomerization and execution of necroptosis. RIPK1 kinase activity also promotes cell-autonomous activation of proinflammatory cytokine production in necroptosis. However, the signaling pathways downstream of RIPK1 kinase in necroptosis and how RIPK1 kinase activation controls inflammatory response induced by necroptosis are still largely unknown. Here, we quantitatively measured the temporal dynamics of over 7000 confident phosphorylation-sites during necroptosis using mass spectrometry. Our study defined a RIPK1-dependent phosphorylation pattern in late necroptosis that is associated with a proinflammatory component marked by p-S473 TRIM28. We show that the activation of p38 MAPK mediated by oligomerized MLKL promotes the phosphorylation of S473 TRIM28, which in turn mediates inflammation during late necroptosis. Taken together, our study illustrates a mechanism by which p38 MAPK may be activated by oligomerized MLKL to promote inflammation in necroptosis.

## Introduction

Necroptosis is a form of regulated necrosis that can be activated in apoptosis-deficient conditions. RIPK1 kinase activity is critical for regulating necroptosis and cell-autonomous cytokine production during necroptosis [1, 2]. Necrostatin-1s (Nec-1s), the first small molecule inhibitor of RIPK1 kinase developed, is an effective inhibitor of necroptosis and inflammation [1, 3, 4]. Necroptosis and RIPK1 kinase-dependent inflammation have been implicated in pathogenesis of various human diseases, including inflammatory bowel disease, liver cancer, and neurodegenerative diseases [5]. Hallmarks of necroptosis including the phosphorylation of RIPK1 and elevated levels of insoluble RIPK1, RIPK3 and MLKL are found in post-mortem human pathological samples such as patients with amyotrophic lateral sclerosis (ALS) and Alzheimer’s disease (AD) [6–8]. Inhibition of RIPK1 using Nec-1s and other small molecule inhibitors of RIPK1 has shown efficacy in various animal models of human diseases [3, 4, 9–12]. RIPK1 inhibitors have been advanced into human clinical studies for the treatment of human inflammatory and degenerative diseases including inflammatory bowel diseases, rheumatoid arthritis, psoriasis, ALS and AD [5]. Thus, understanding the mechanism in which RIPK1 kinase controls necroptosis and necroptosis-associated inflammation may help to develop improved strategies and biomarkers for the treatment of human diseases involving RIPK1 activation.

TNFα, an important proinflammatory cytokine implicated in a multitude of human diseases, is one of the most extensively studied triggers of necroptosis [13]. In TNFα-stimulating cells, RIPK1 is rapidly recruited into complex I that is transiently formed in association with activated TNFR1 [14]. Under certain specific cellular conditions, RIPK1 kinase may be activated in complex I [15]. Activated RIPK1 in turn mediates the formation of RIPK1/RIPK3/MLKL complex (complex IIb or necrosome), which promotes the oligomerization of MLKL and execution of necroptosis [16–18]. The activation of RIPK1 and formation of necrosome robustly promote the sustained activation of NF-κB and MAPK to mediate cell-autonomous proinflammatory cytokine production [1]. However, the downstream phosphorylation signaling events regulated by RIPK1 kinase that promote proinflammatory cytokine production remain poorly understood.

TRIM28, a large multi-domain protein (110 kDa), is a member of human Tripartite motif-containing (TRIM) protein family and also known as KAP1 (Krüppel-Associated Box (KRAB)-Associated Protein 1) or TIF1-β (Transcriptional Intermediary Factor 1β) [19]. TRIM28 is known to be phosphorylated in response to a variety of extracellular stresses such as DNA damage to attenuate its binding with heterochromatin protein 1 (HP1), which results in transcriptional activation of target genes to promote DNA repair and cell survival [20]. TRIM28 is involved in regulating endothelial inflammatory responses by maintaining expression of TNF receptors [21]. p38 MAP kinases, including p38-α (MAPK14), -β (MAPK11), -γ (MAPK12/ERK6), and -δ (MAPK13), are members of mitogen-activated protein (MAP) kinase family [22] and essential regulators of MAP kinase transduction cascades mediated by proinflammatory cytokines and environmental stress [23]. Inhibition of p38 has been shown to inhibit the transcription of proinflammatory cytokines in necroptosis [1]. However, it is unclear how p38 is activated in necroptosis, nor do we know the involvement of TRIM28 or its phosphorylation in necroptosis.

In the present study, we conducted a systemic quantitative analysis of phosphoproteome regulated by RIPK1 kinase across a time course from complex I to complex IIb formation during necroptosis of FADD-deficient Jurkat cells induced by TNFα. Our study identified a prominent RIPK1-dependent phosphorylation component in the global phosphoproteome in late-stage of necroptosis, which was highly enriched in the pathways of gene expression and inflammatory response. We characterized an example of such late-stage phosphorylation events, the phosphorylation of S473 TRIM28, and showed the activation of p38 during necroptosis induced by its interaction with oligomerized MLKL mediates this phosphorylation event. Finally, we found that the phosphorylation of Ser473 TRIM28 promoted the production of proinflammatory cytokines in necroptosis. Since the activation and oligomerization of MLKL is mediated by the interacting RIPK1 and RIPK3 in amyloid conformation during necroptosis, our study may provide an example as how functional amyloids promote inflammation.

## Results

### Quantitative analysis of RIPK1 kinase-dependent phosphoproteome in necroptosis

Necroptosis of FADD-deficient Jurkat cells are defective upon TNFα [3] began ~4 h after addition of TNFα, which was effectively inhibited by Nec-1s (Fig. S1A). Activation of RIPK1 kinase, as indicated by its activation biomarker p-S166 RIPK1 [24], was detectable within 15 min of TNFα addition and peaked at 2–4 h and effectively blocked by Nec-1s (Fig. S1B).

Quantitative mass spectrometry analysis was performed at indicated time points (Fig. S1C). We identified 8722 phosphorylation sites with localization probability over 75% confidence including 153 novel sites previously unidentified, and quantitatively measured 7045 phosphosites (Table S1). Most of peptides have coefficient of variations (CVs) among 6 replicates below 25% (Fig. S1D). Total proteome analysis identified 4448 proteins and 41974 peptides with FDR lower than 1%. Among those, 3315 proteins were quantified in at least two replicates across all the time points (Table S2). The log_2_Ratio distribution of observed peptides used for quantification of phosphosites and proteome is normal, and more changes were found at the phosphosites levels than at protein levels (Fig. S1E), suggesting that the changes of phosphosites are most likely independent from the changes in protein abundance.

**Fig. 1:**
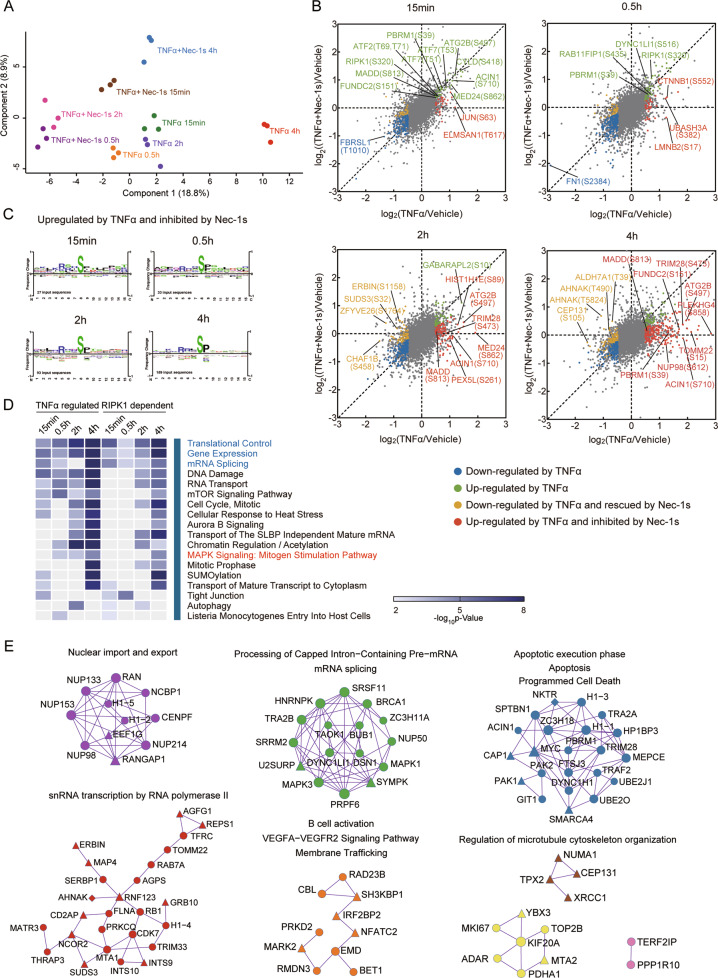
Quantitative analysis of necroptosis phosphoproteome in TNFα-stimulated FADD-deficient Jurkat cells. **A** PCA plot based on log_2_Ratios of FADD-deficient Jurkat cells treated with TNFα vs. that of vehicle only or (TNFα + Nec-1s) vs. that of vehicle only at the indicated time points of treatments. **B** Scatter plots comparing log_2_Ratios of FADD-deficient Jurkat cells treated with TNFα vs. that with vehicle only and FADD-deficient Jurkat cells treated with (TNFα + Nec-1s) vs. with vehicle only. The green and blue dots represent phosphosites with log_2_Ratios of FADD-deficient Jurkat cells treated with TNFα vs. that with vehicle only, either ≥0.485 or ≤ -0.485 and *p-*value < 0.05, respectively, but were not affected by the addition of Nec-1s. The phosphosites that were affected by treatment with TNFα and inhibited or rescued over 50% by Nec-1s were colored by red or yellow, respectively. **C** The consensus motif analysis of phosphorylation sites that are dependent on RIPK1 kinase activation. The pre-aligned sequences of 15 residues or less containing phosphosites upregulated by TNFα and inhibited by treatment with Nec-1s were subjected to motif analysis using the tool in PhosphositePlus^®^. **D** Pathway analysis of phosphoproteins. Phosphosites that were up or downregulated in TNFα-treated cells, which were inhibited or rescued by Nec-1s were subjected to GO analysis. The heat map displays a cluster of significantly enriched pathways. The color scale is defined based on –log_10_ (*p*-value). The pathways highlighted in red and blue are corresponding to those related to inflammation and gene expression, respectively. **E** Network analysis of 247 phosphoproteins containing 299 phosphosites, which were up- or downregulated by TNFα and inhibited or rescued by Nec-1s at the time point of 4 h. MCODE algorithm was applied to identify the neighborhoods where proteins were densely connected. Each MCODE network is assigned a unique color. GO enrichment analysis was applied to each MCODE network to assign biological functions to the network component. Circles, triangles and diamonds represent proteins containing Nec-1s inhibited, rescued and both inhibited and rescued phosphosites, respectively. TNFα, 20 ng/mL; Nec-1s, 10 μM.

A principal component analysis (PCA) shows that the phosphoproteomes of replicates at each time point after treatments of TNFα or TNFα + Nec-1s are clustered tightly, demonstrating the good reproducibility of the measurements (Fig. 1A). Interestingly, the phosphoproteomes stimulated by TNFα are clearly distinct from that treated with TNFα plus Nec-1s at all time points analyzed. In addition, the phosphoproteomes at early time points after the addition of TNFα or TNFα + Nec-1s at 15 min and 0.5 and 2 h show distinctions from that of late time point at 4 h (Fig. 1A), demonstrating the temporal dynamics in TNFα-induced and RIPK1-dependent phosphoproteomic changes during necroptosis. From these results, we conclude that the overall phosphoproteomes stimulated by TNFα vs. that of TNFα + Nec-1s are substantially different, indicating a significant RIPK1-dependent phosphorylation component in the global phosphoproteome of FADD-dependent Jurkat cells stimulated by TNFα.

As shown in the volcano plots (Fig. S2A), we defined the phosphosites with log_2_Ratios ≥ 0.485 or ≤ −0.485 and *p-*value < 0.05 as significantly upregulated or downregulated by TNFα stimulation, respectively. Several known phosphosites on proteins in response to TNFα stimulation at 15 min were found to be significantly increased, including Ser418-CYLD, Thr53-ATF7, T69/T71-ATF2 and Ser320-RIPK1 [25], which were not affected by the treatment with Nec-1s, providing a validation for this study. We defined the phosphosites that were inhibited or rescued over 50% by Nec-1s as regulated by RIPK1 kinase (Figs. 1B and S2B). Notably, the pattern of phosphoproteome during necroptosis shows temporal specific features (Fig. 1B), and consistent with the dynamic changes of RIPK1 kinase activation during necroptosis shown in Fig. S1B. Nec-1s treatment exerts increasingly prominent effects on the phosphoproteome at late stage of necroptosis (Fig. 1B and Fig. S2B, C). For example, the increased levels of p-S473 TRIM28, p-S612 NUP98 and p-S15 TOMM22 found only at the late necroptosis were suppressed in Nec-1s treated samples. Some phosphosites, such as p-S710 ACIN1, p-S813 MADD and p-S497 ATG2B, were upregulated by TNFα stimulation at 15 min, 2 h and 4 h, but were only inhibited by Nec-1s at 2 h and 4 h, indicating that those phosphosites might be regulated distinctly in the early and late stage of necroptosis (Fig. 1B). The analysis of sequence consensus motif for phosphosites upregulated by TNFα that were inhibited by Nec-1s showed that RXXpSP motif was significantly enriched at all four time points (Fig. 1C).

The regulated pathways in the early time points (15 min, 0.5 and 2 h) of TNFα-treated FADD-deficient Jurkat cells are distinguishable from that with TNFα + Nec-1s, and while at late 4 h time point, there were considerable convergence in the regulated pathways, suggesting the dominance of RIPK1-dependent regulation in necroptosis with prolonged TNFα treatment (Fig. 1D). Significant enrichments of the pathways relevant to gene expression and MAPK signaling at late time points were affected by Nec-1s (Fig. 1D). These results highlight the role of RIPK1 kinase in mediating signal transduction with prolonged TNFα stimulation during necroptosis.

Interestingly, using MCODE algorithm we identified substantial enrichments of seven protein complexes that were regulated in RIPK1-dependent manner during late necroptosis (Fig. 1E and Table S3). In addition to the genes involved in the apoptotic execution, the RIPK1-dependent phosphoproteome is enriched with the complexes involved in the nuclear import and export, mRNA splicing, B-cell activation, cytoskeleton organization, and snRNA transcription (Fig. 1E and Table S3).

### RIPK1-dependent phosphorylation of S473-TRIM28 during late necroptosis

**Fig. 2:**
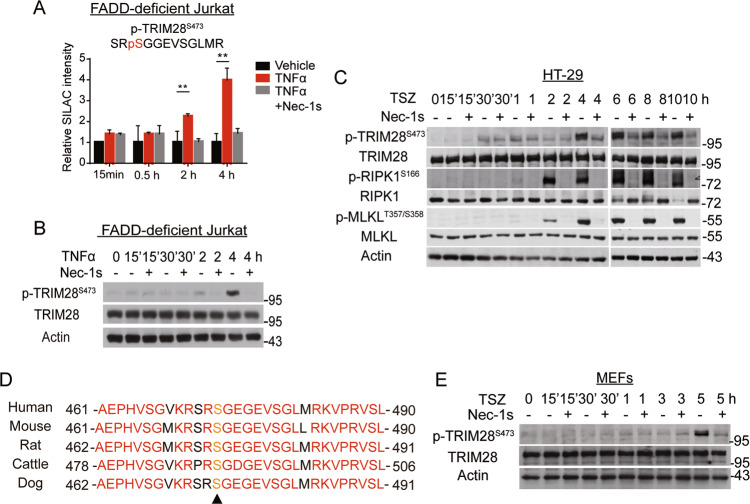
TRIM28 is phosphorylated at Ser473 in necroptosis. **A** Relative SILAC intensities of TRIM28 S473 phosphorylated peptides in cells treated with vehicle only, TNFα and TNFα + Nec-1s at different time points as indicated. **B** FADD-deficient Jurkat cells were treated with TNFα and TNFα + Nec-1s as indicated. The total protein levels of TRIM28 and phosphorylation levels of TRIM28 S473 were analyzed by western blotting. **C** HT-29 cells were treated with TNFα + SM164 + zVAD (TSZ) at indicated time points in the presence or absence of Nec-1s. The cell lysates were analyzed by western blotting with indicated antibodies. **D** Alignment of amino acid sequences of TRIM28 around Ser473 in mammalian species. **E** MEFs were treated with TSZ at indicated time course in the presence or absence of Nec-1s. The cell lysates were analyzed by western blotting using antibodies against total TRIM28 and phosphorylation of TRIM28 S473. TNFα, 20 ng/mL; zVAD, 25 μM; SM164, 50 nM; Nec-1s, 10 μM. Data were presented as mean ± SEM. **t-test *p* < 0.01, *n* = 6.

We next focused on Ser473 TRIM28, one of the most robustly phosphorylated sites in response to TNFα and was inhibited by Nec-1s (Fig. 2A). Validated by western blotting, the phosphorylation of S473 TRIM28 in FADD-deficient Jurkat cells was elevated at 2 h and further boosted at 4 h after TNFα addition, which was obviously inhibited by Nec-1s (Fig. 2B). We found that the phosphorylation of S473 TRIM28 was also detectable in necroptotic HT-29 cells induced by TNFα/SM164/zVAD (TSZ). The phosphorylation of S473 TRIM28 was slightly upregulated at early time points between 30 min and 2 h with TSZ treatment, which was not inhibited by Nec-1s. In contrast, the phosphorylation of S473 TRIM28 induced at late time points from 4 h to 10 h with TSZ stimulation was inhibited by Nec-1s (Fig. 2C). Thus, the phosphorylation of S473 TRIM28 was regulated in RIPK1-independent manner in early time points and RIPK1-dependent manner in late time points during necroptosis.

The phosphorylation of S473 TRIM28 was detected in HT-29 cells treated with TNFα alone in early time points from 30 min to 2 h, which was not inhibited by Nec-1s (Fig. S3A). In HT-29 cells treated with TNFα/SM164 (TS), the phosphorylation of S473 TRIM28 was detected from 30 min until 4 h but was not inhibited by Nec-1s (Fig. S3B). These results suggest that RIPK1-dependent phosphorylation of S473 TRIM28 in necroptosis is mechanistically different from that induced by TNFα only and TS.

In addition, we found that the phosphorylation of S473 TRIM28 was also detected in necroptosis induced by treatment with TNFα/CHX/zVAD (TCZ) but cannot be inhibited by Nec-1s (Fig. S3C). However, treatment with cycloheximide alone could induce the phosphorylation of S473 TRIM28 (Fig. S3D). Thus, the increased phosphorylation of S473 TRIM28 induced by cycloheximide is likely to mask the RIPK1 kinase regulated phosphorylation of S473 TRIM28 in necroptosis induced by TCZ.

S473 and S824 TIRM28 are phosphorylated by ATM/ATR kinases and Chk1 in response to DNA damage [20, 26]. As shown in Fig. S4A, C and D, treatment with etoposide, a DNA damage inducer, efficiently induced phosphorylation of S473 TRIM28 rather than S824 that was not inhibited by Nec-1s, RIPK3 inhibitor GSK’872, MLKL inhibitor NSA or Chk1 inhibitor, nor was RIPK1 kinase activation induced by etoposide. The phosphorylation of H2A.X S139, a marker for DNA damage, was not detected across the time course of TSZ stimulation in these cells (Fig. S4B). These results indicate that the regulation of S473 TRIM28 phosphorylation in necroptosis is distinct from that induced by DNA damage.

S473 TRIM28 is a residue highly conserved in its orthologues among mammalian species, including human, mouse, rat, cattle, and dog (Fig. 2D). Consistently, phosphorylation of S473 TRIM28 was induced at 5 h after TSZ stimulation, which was inhibited by treatment with Nec-1s in mouse embryonic fibroblasts (MEFs) (Fig. 2E).

Taken together, we demonstrate that the phosphorylation of S473 TRIM28 is induced in late stage of necroptosis in RIPK1-dependent manner in both human and mouse cells, but is mediated by a mechanism distinct from that of apoptosis and DNA damage.

### TRIM28 interacts with MLKL and S473 TRIM28 is phosphorylated in complex IIb

**Fig. 3:**
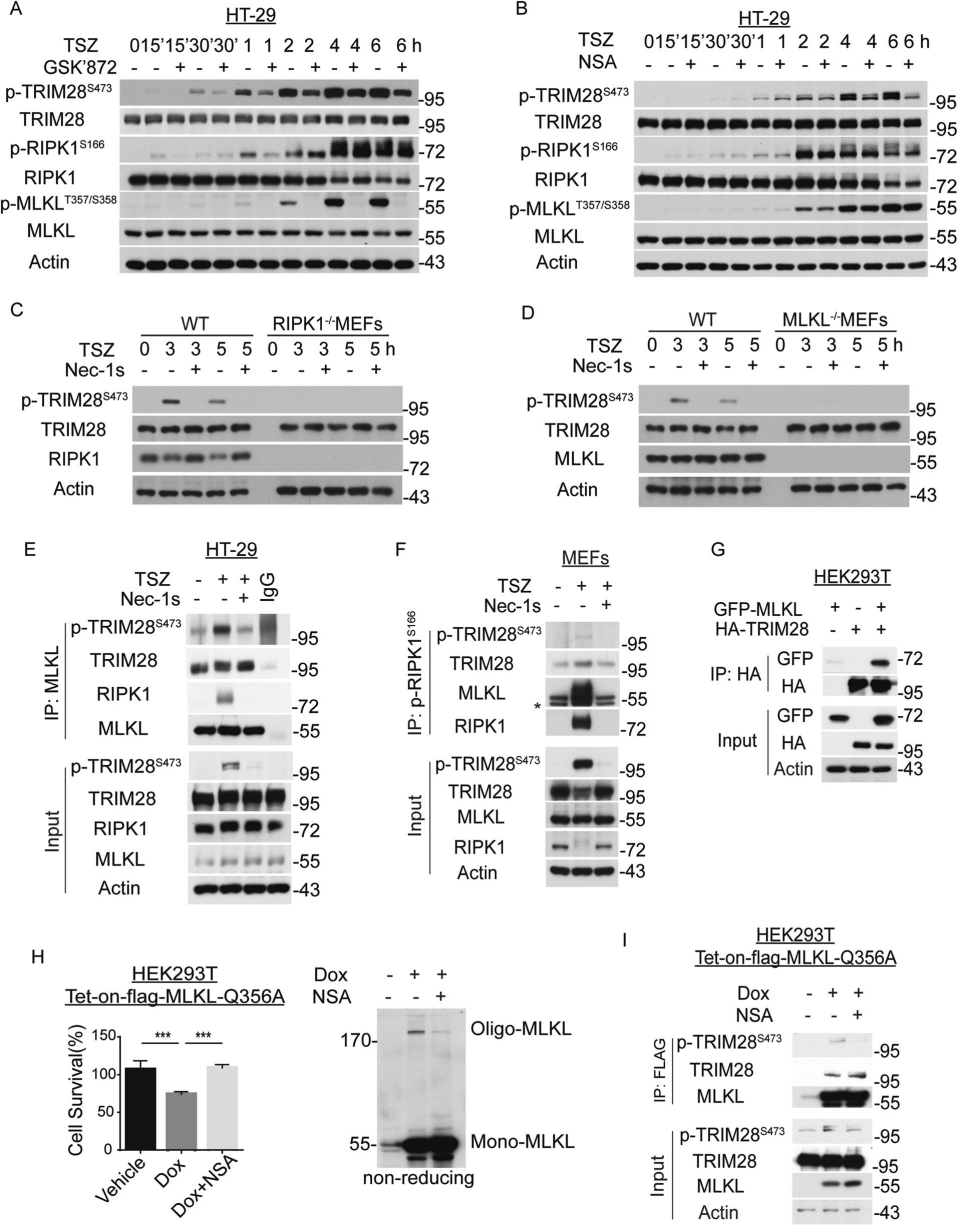
TRIM28 interacts with MLKL and is phosphorylated at Ser473 in complex IIb during necroptosis. **A**, **B** HT-29 cells were treated with the compounds as indicated. The levels of specific proteins were analyzed by western blotting using indicated antibodies. **C**, **D** RIPK1^−^^/−^ (**C**) and MLKL^−/−^ (**D**) MEFs were treated with TSZ as indicated. The levels of total TRIM28 and phosphorylation of TRIM28 S473 were analyzed by western blotting. **E** HT-29 cells were treated with TSZ or TSZ + Nec-1s for 4 h. Lysates were immunoprecipitated with MLKL antibody and analyzed by western blotting using indicated antibodies. **F** MEFs were treated with TSZ for 3 h. The cell lysates were immunoprecipitated by pRIPK1^S166^ antibody. Both lysate input and immunoprecipitation samples were analyzed by western blotting using indicated antibodies. *, non-specific bands. **G** 293T cells were transfected with GFP-MLKL and HA-TRIM28 for 24 h. The cell lysates were immunoprecipitated with anti-HA resin and analyzed by western blotting using indicated antibodies. **H** 293T cells were transfected with tet-inducible flag-tagged MLKL Q356A expression vector. MLKL oligomerization was induced by doxycycline with or without NSA. Cell viability was determined by CellTiter-Glo (left). Cell lysates were separated by non-reducing SDS/PAGE and analyzed by western blotting using MLKL antibody (right). **I** 293T cells were transfected with tet-inducible flag-tagged MLKL Q356A expression vector. After doxycycline induction, the cell lysates were prepared and immunoprecipitated by anti-Flag resin and analyzed by western blotting using indicating antibodies. TNFα, 20 ng/mL; zVAD, 25 μM; SM164, 50 nM; Nec-1s, 10 μM; GSK’872, 10 μM; NSA, 2 μM; Dox, 2 ng/mL. Data were presented as mean ± SEM. ***t-test *p* < 0.001, *n* = 3.

We next investigated how TRIM28 may be phosphorylated in late necroptosis in HT-29 cells and MEFs. The phosphorylation of S473 TRIM28 induced by TSZ was inhibited by RIPK3 inhibitor GSK872 and MLKL inhibitor NSA without affecting RIPK1 kinase activation, and totally abolished in RIPK1^−/−^ and MLKL^−^^/−^ MEFs (Fig. 3A–D). These results suggest that the phosphorylation of S473 TRIM28 is likely mediated by the activation of MLKL, which is dependent upon RIPK1 activation and the formation of complex IIb.

We found that TRIM28 was constitutively bound with MLKL in control and necroptotic cells and p-S473 TRIM28 was also in complex with MLKL (Fig. 3E). Treatment with Nec-1s inhibited the phosphorylation of S473 TRIM28 but did not affect its binding with MLKL. We also detected enhanced interaction of p-S473 TRIM28 with activated RIPK1 (Fig. 3F). We further confirmed the interaction of TRIM28 with MLKL by co-overexpression in 293 T cells (Fig. 3G) and the pseudo-kinase domain of MLKL mediates its binding with TRIM28 (Fig. S4E–G). Thus, TRIM28 interacts with MLKL in unstimulated cells and p-S473 TRIM28 is a part of complex IIb during necroptosis.

MLKL Q356A mutant can spontaneously aggregate and induce cell death [27]. Interestingly, we found that TRIM28 was recruited to oligomerized MLKL Q356A upon the induction of expression, and the phosphorylation levels of S473 TRIM28 associated with MLKL Q356 was inhibited by NSA (Fig. 3H, I). Taken together, we demonstrate that TRIM28 interacts with MLKL and is phosphorylated at S473 in complex IIb downstream of MLKL oligomerization during necroptosis.

### p38 activation induced by MLKL oligomerization mediates S473 TRIM28 phosphorylation in necroptosis

**Fig. 4:**
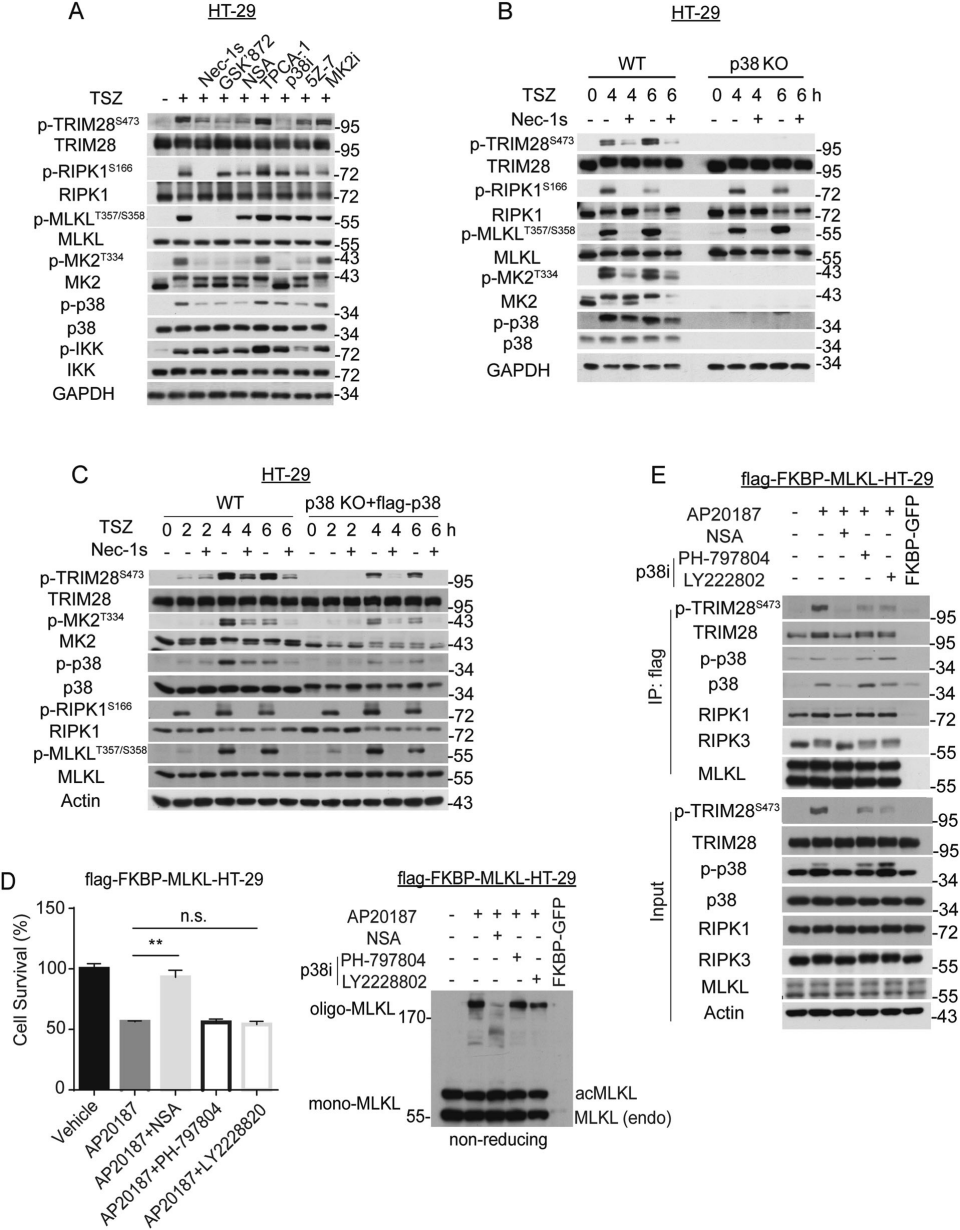
MLKL oligomerization induces p38 activation to mediate the phosphorylation of Ser473 TRIM28. **A** HT-29 cells were treated with the indicated inhibitors followed by TSZ treatment for 4 h. The cell lysates were analyzed by western blotting using indicated antibodies. **B** p38 KO cells were engineered by CRISPR–Cas9 technology and p38 expressing vector was reconstituted in HT-29 cells. WT and p38 KO HT-29 cells were treated with TSZ in the presence or absence of Nec-1s at indicated time points. The cell lysates were analyzed by western blotting using indicated antibodies. **C** WT and p38 reconstituted p38 KO HT-29 cells were treated with TSZ in the presence or absence of Nec-1s as indicated. The cell lysates were analyzed by western blotting using indicated antibodies. **D**, **E** HT-29 cells were transfected with an expression vector of flag-tagged MLKL fused with two AP20187-binding (FKBPv) domains. The cells were pretreated with indicated compounds for 4 h followed by treatment with AP20187 to induce MLKL oligomerization. Cell viability was determined by CellTiter-Glo after treatment with AP20187 for 2 h (D Left). Cell lysates were separated by non-reducing SDS/PAGE and analyzed by western blotting using MLKL antibody (D Right). The cell lysates were immunoprecipitated by anti-Flag resin after treatment with AP20187 for 2 h and analyzed by western blotting using indicated antibodies (E). acMLKL, oligomerizable MLKL; endo, endogenous. TNFα, 20 ng/mL; zVAD, 25 μM; SM164, 50 nM; Nec-1s, 10 μM; NSA, 4 μM; TPCA-1, 10 μM; p38i PH-797804, 10 μM; p38i LY2228820, 4 μM; 5Z-7, 500 nM; MK2i MK2-IN-1, 10 μM; AP20187, 20 μM. Data were presented as mean ± SEM. **t-test *p* < 0.01, *n* = 3. n.s., not significant.

Next, we explored the mechanism that mediates the phosphorylation of S473 TRIM28 in complex IIb. Using mass spectrometry, we identified several kinases, including STK4, WNK1, PRKCD, PRKCI and ADK, bound to oligomerized MLKL after Dox induction of expression of MLKL Q356A and the addition of NSA inhibited the binding (Table S4). However, knocking down any of those candidate kinases did not affect the phosphorylation of S473 TRIM28 in necroptosis (Fig. S5A). Strikingly, treatment with p38 inhibitor abolished the phosphorylation of S473 TRIM28 in HT-29 cells induced by TSZ but IKK and MK2 inhibitors had no effect, and TAK1 inhibitor 5Z-7 partially inhibited TRIM28 phosphorylation (Fig. 4A). p38 knockout abolished the phosphorylation of S473 TRIM28 compared to that of WT HT-29 cells during necroptosis induced by TSZ (Fig. 4B, C).

Furthermore, we transfected an inducible MLKL dimerization construct with fused FKBP at C terminus of MLKL into HT-29 cells. The addition of AP20187 effectively induced MLKL oligomerization and cell death, which can be inhibited by NSA; p38 inhibitors had no effect on cell death induced by MLKL oligomerization (Fig. 4D). Consistently, interaction of TRIM28 with MLKL and phosphorylation of S473 TRIM28 was induced by MLKL oligomerization, which was inhibited by the treatment with NSA and p38 inhibitors (Fig. 4E). Finally, we expressed flag-FKBP-MLKL in WT and RIPK3 knockdown HT29 cells. Induction of MLKL dimerization by AP20187 is sufficient to activate p-S473 TRIM28 in both WT and RIPK3-kd HT29 cells. Knockdown of RIPK3 even slightly increased the p-S473 TRIM28 (Fig. S5B). Taken together, these results suggest that phosphorylation of S473 TRIM28 in necroptosis is mediated by p38, which is activated upon binding with oligomerized MLKL.

TAK1-MKK3 axis can activate p38 and subsequently result in MK2 phosphorylation downstream of TNFR1 and TLRs pathways [22]. Treatment with 5Z-7 robustly inhibited p38 activation and phosphorylation of S473 TRIM28 during early time points (0.5–2 h) after induction of necroptosis when phosphorylation of MLKL was not yet detected; treatment with Nec-1s had no effect on either p38 activation or phosphorylation of S473 TRIM28 during the early time points (Fig. S5C). In contrast, p38 activation and phosphorylation of S473 TRIM28 at late time points (4–6 h) during necroptosis were inhibited by Nec-1s but not 5Z-7 (Fig. S5C). In addition, knockout of MKK3 or MK2 had no effect on p38 activation or phosphorylation of S473 TRIM28 during necroptosis (Fig. S5D, E). We also found that knockdown of both MKK3/MMK6 had no effect on p-p38 or p-S473-TRIM28 (Fig. S5F). Taken together, these results suggest that p38 activation and phosphorylation of S473 TRIM28 during necroptosis is mediated by two mechanisms, the first mechanism is activated before MLKL activation and is TAK1-dependent and RIPK1-independent, while the second mechanism is TAK1 independent and is dependent upon RIPK1 and MLKL activation.

### Phosphorylation of S473 TRIM28 regulates cytokine production in necroptosis

We next examined the effect of S473 TRIM28 phosphorylation on necroptosis. The cell death and cell viability assays showed that TRIM28 KO had no effects on cell death induced by TSZ in HT-29 cells (Fig. S6A–D). The cell death assays showed that TRIM28 KO promoted cell death 10 h after stimulation of TNFα alone but had no effects on cell death induced by TS or TC (TNFα/CHX) in FADD-deficient Jurkat cells (Fig. S6E–G). However, there was no obvious effect on cell death in both HT-29 and FADD-deficient Jurkat cells reconstituted with TRIM28 S473 mutations by indicated stimulations of TSZ, TNFα only or TS compared to that of WT cells (Fig. S6C–F). Moreover, TRIM28 knockdown and TRIM28 S473 mutations did not affect the oligomerization and phosphorylation of MLKL in necroptosis (Fig. S6H–J). These results suggest that phosphorylation of S473 TRIM28 is an event downstream of MLKL.

**Fig. 5:**
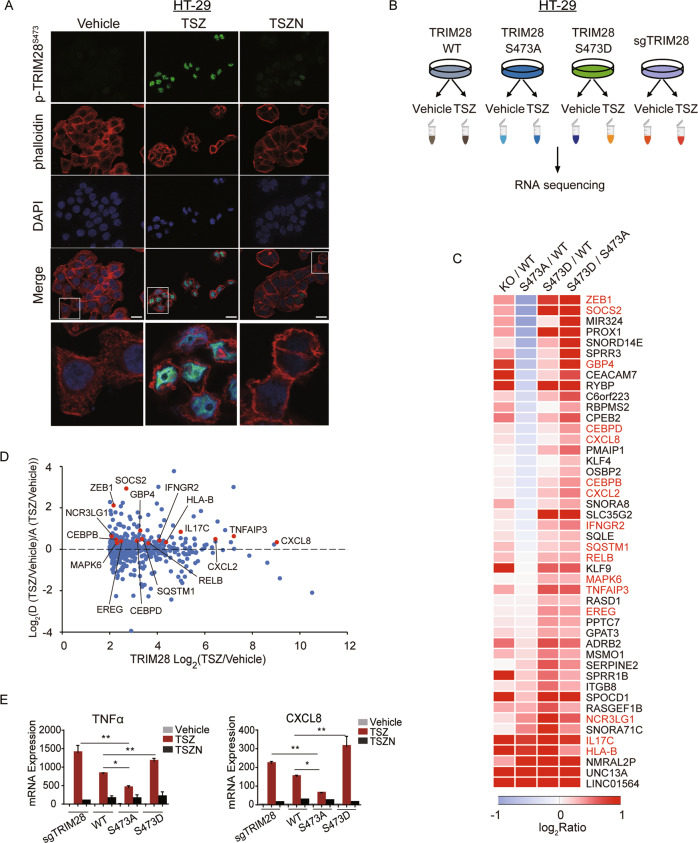
Phosphorylation of S473 TRIM28 regulates cytokine production in necroptosis. **A** Immunostaining of p-S473 TRIM28, phalloidin and DAPI in HT-29 cells treated with TSZ for 8 h in the presence or absence of Nec-1s as indicated. **B** The scheme for RNAseq analysis of TRIM28 KO HT-29 cells and TRIM28 WT, S473A, and S473D reconstituted TRIM28 KO HT-29 cells treated with TSZ. Cells were treated with TSZ or vehicle only for 8 h, respectively. **C** 46 genes upregulated in TRIM28 KO HT29 cells and TRIM28 S473D reconstituted TRIM28 KO HT29 cells compared to that of TRIM28 WT reconstituted TRIM28 KO HT29 cells by TSZ induction were shown in the heat map. Items highlighted in red were genes involved in inflammatory signaling pathways. **D** Scatter plot comparing log_2_Ratios of TRIM28 WT reconstituted TRIM28 KO HT29 cells treated with TSZ vs. that with vehicle only and log_2_Ratios of TRIM28 S473D reconstituted TRIM28 KO HT29 cells treated with TSZ vs. TRIM28 S473A reconstituted TRIM28 KO HT29 cells treated with TSZ. **E** TRIM28 KO HT29 cells or TRIM28 WT, S473A, or S473D reconstituted TRIM28 KO HT29 cells were treated with TSZ for 8 h in the presence or absence of Nec-1s. The mRNA levels of TNFα and CXCL8 were measured by qPCR. Data were presented as mean ± SEM. **t-test *p* < 0.01, *n* = 3. TNFα, 20 ng/mL; zVAD, 25 μM; SM164, 50 nM; Nec-1s, 10 μM.

TRIM28 is localized in nucleus and acts as a transcription repressor, and phosphorylation of TRIM28 S473 is known to promote gene transcription [28]. We demonstrated that phosphorylated TRIM28 was present in nucleus and co-localized with DAPI in necroptotic cells by immunofluorescence (Fig. 5A). RNAseq analysis showed that the expression of 536 genes was upregulated and that of 380 genes downregulated with *p-*value < 0.05 and log_2_Ratio > 2 or < -2 by TSZ stimulation in TRIM28 WT reconstituted TRIM28 KO HT29 cells (Fig. 5B, Fig. S7A, and Table S5). Among those 536 TSZ-upregulated genes, the expression levels of 193 genes were further upregulated in TRIM28 KO cells and among them, the expression of 133 genes was upregulated in both TRIM28 KO and TRIM28 S473D reconstituted TRIM28 KO cells compared to that of WT TRIM28 reconstituted cells (log_2_Ratios > 0). Among these 133 genes, the expression of 88 genes was upregulated by TRIM28 S473D compared to TRIM28 S473A (log_2_Ratio > 0), and the expression levels of 46 genes have increased log_2_Ratios > 0.26 as shown in the heat map (Fig. 5C). The expression of several genes in inflammatory signaling pathways (highlighted in Fig. 5C), such as CXCL8, IL17C, RELB, MAPK6, TNFAIP3, IFNGR2, SQSTM1, was upregulated at the transcriptional levels in necroptosis and enhanced by TRIM28 KO and the expression of TRIM28 S473D while inhibited by the expression of TRIM28 S473A (Fig. 5D). Moreover, the mRNA expression levels of TNFα and CXCL8 examined by qPCR were dramatically increased in HT-29 and TRIM28 WT reconstituted HT-29 cells by induction of necroptosis, further enhanced by TRIM28 KO and TRIM28 S473D and suppressed by TRIM28 S473A (Fig. 5E). Taken together, we demonstrate that phosphorylation of S473 TRIM28 during necroptosis is involved in regulating transcription of genes associated with inflammation.

## Discussion

**Fig. 6:**
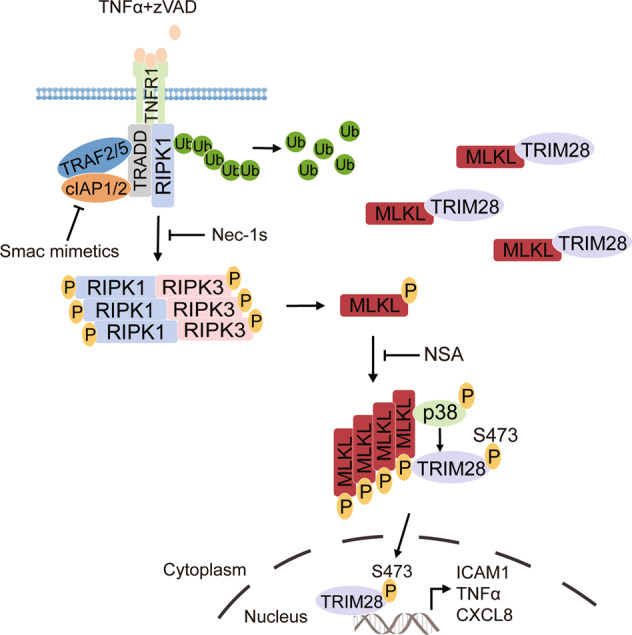
A schematic model for phosphorylation of Ser473 TRIM28 mediated by p38 MAPK in regulating cytokine production in necroptosis. The activation of RIPK1 kinase promotes its interaction with RIPK3 to form complex IIb (necrosome), which leads to the activation of RIPK3. The activated RIPK3 in turn mediates the phosphorylation of MLKL to promote its oligomerization. TRIM28 constitutively interacts with MLKL in control condition. Oligomerized MLKL interacts with p38, which promotes the activation of p38. The activated p38 in turn mediates the phosphorylation of Ser473 TRIM28. The Ser473 phosphorylated TRIM28 promotes proinflammatory cytokine production.

We present a quantitative phosphoproteomic study that reveals the dynamic time-dependent changes of RIPK1 kinase-dependent phosphoproteome during necroptosis induced by TNFα in FADD-deficient Jurkat cells. Our study reveals a set of RIPK1-dependent phosphorylation events in late necroptosis. The binding of p38 with oligomerized MLKL mediates a set of late phosphorylation events that includes the phosphorylation of TRIM28. Our results suggest that the RIPK1-dependent phosphorylation events in late necroptosis may be mediated by the downstream events of RIPK1-dependent signaling. Consistently, the sequence consensus motif for Nec-1s inhibited phosphosites found in this study was RXXpSP, which is similar to that of pSP sequence motif previously reported for RIPK3-dependent phosphoproteome [29]. Furthermore, since we found that the formation of necrosome and oligomerization of MLKL could promote the activation of p38 MAPK, which mediates the phosphorylation of substrates with the sequence consensus motif of pT/pSP [30], the RXXpSP motif found by this study may also reflect the activation of MAPK during necroptosis. Since the interacting RIPK1 and RIPK3 in necrosome (complex IIb) may be present in the conformation of amyloid fibers and phosphorylation of MLKL by RIPK3 promotes the oligomerization of MLKL [31], our results suggest the possibility that p38 activation in binding with MLKL is sensitive to its conformation and is activated by the oligomerized MLKL, which in turn mediates the phosphorylation of S473 TRIM28 to promote the transcription of proinflammatory cytokines (Fig. 6). Thus, the activation of p38 and phosphorylation of S473 TRIM28 may provide a mechanism by which functional amyloid fibers promote inflammatory response. In addition, our analysis also demonstrated a significant enrichment of the proteins involved in gene transcription, splicing and nuclear imports whose phosphorylation were regulated in RIPK1-dependent manner during late-stage necroptosis. Thus, it is possible that there is a coordinated activation of proinflammatory responses in necroptosis at multiple levels of gene expression.

p38 MAPKs have established roles as key signaling molecules that regulate the production of proinflammatory cytokines in response to environmental stresses and are implicated in the pathogenesis of many inflammatory-driven conditions [32]. Therapeutic inhibition of p38 MAPKs for attenuating inflammation has been investigated in the last 3 decades. The therapeutic potential of p38 MAPK inhibitors was initially explored in clinical trials for the treatment of human inflammatory conditions such as rheumatoid arthritis and Crohn’s disease, but the progression of such studies was prevented due to poor clinical efficacy and unacceptable side effects. Our study demonstrates the role of RIPK1 activation in promoting the activation of p38 in mediating inflammation activated by necroptosis. RIPK1 inhibitors have been advanced into multiple of human clinical studies beyond Phase I including for the treatment of rheumatoid arthritis and Crohn’s disease [5]. Thus, targeting RIPK1 might provide an alternative means to inhibit p38-mediated inflammatory signaling.

Here, we describe a dual role of TAK1 and RIPK1 in regulating the phosphorylation of S473 TRIM28 during necroptosis. TAK1 (TGF-β activated kinase 1) is a MAPK kinase kinase family serine threonine kinase that is known to be involved in the signaling cascades involving p38 [33]. TAK1 activation has also been shown to mediate the phosphorylation of S321 mRIPK1/S320 hRIPK1 during the early stage of necroptosis [34]. Since the early TAK1-dependent phosphorylation of S473 TRIM28 during necroptosis is also inhibited by p38 KO and p38 inhibitors, TAK1 may cooperate with RIPK1-RIPK3-MLKL axis to promote inflammatory response activated by necroptosis. Since TAK1 can exert an inhibitory activity on RIPK1 [34], it is conceivable that the activation of TAK1 may inhibit the subsequent RIPK1 activation under certain condition to reduce inflammation. Overall, we delineate a landscape of dynamic phosphoproteomes downstream of RIPK1 kinase activation during necroptosis and reveal the mechanism by which p-S473 TRIM28, one of the most upregulated phosphosites in necroptosis, is controlled and its function in promoting inflammation. Our work provides insights into how RIPK1 kinase activation may regulate downstream phosphorylation cascades to promote inflammation under chronic inflammatory conditions.

## Materials and methods

### Reagents

The following commercial reagents were used in this study: TNFα (Novoprotein, C008), 5Z-7-Oxozeaenol (5Z-7) (Sigma-Aldrich, O9890), TPCA-1 (MCE, HY-10074), PH-797804 (MCE, HY-10403), LY2228820 (MCE, HY-13241), MK2i (MCE, HY-12834A), GSK’872 (Calbiochem, 530389), NSA (Necrosulfonamide) (Calbiochem, 480073), MK-8776 (Selleck, SCH900776), PD-407824 (Sigma-Aldrich, PZ0111), BHT (Sigma-Aldrich, 34750), BHA (Sigma-Aldrich, 20021), AP20187 (MCE, HY-13992). R-7-Cl-O-Nec-1(Nec-1s), zVAD.fmk and SM164 were made by custom synthesis. Final concentrations of the compounds used in all experiments: TNFα, 20 ng/mL; zVAD, 25 μM; SM164, 50 nM; Nec-1s, 10 μM.

### Cell culture

HEK293T cells, MEFs, FADD-deficient Jurkat cells, and HT-29 cells were originally obtained from the American Type Culture Collection (ATCC). HEK293T cells and MEFs were cultured in Dulbecco’s modified Eagle’s medium (DMEM; Thermo Fisher Scientific, 11965) with 10% (vol/vol) fetal bovine serum (FBS; Thermo Fisher Scientific, 10082-147). FADD-deficient Jurkat cells were cultured in RPMI 1640 medium deficient in l-lysine and l-arginine with 10% (vol/vol) fetal bovine serum deficient in l-lysine and l-arginine, using stable isotope labeling by amino acids in cell culture (SILAC). Recipes of SILAC were obtained from Pandey Lab. HT-29 cells were cultured in McCoy’s 5A medium (Thermo Fisher Scientific, 16600082) with 10% (vol/vol) FBS. All media were supplemented with 1% (vol/vol) penicillin (100 U/mL) and streptomycin (100 U/mL). All of the mammalian cell lines were maintained at 37 °C with 5% CO_2_. The cells were tested routinely using a TransDetect PCR Mycoplasma Detection Kit (Transgen Biotech, FM311-01) to ensure that they were mycoplasma free.

### Generation of knockdown and knockout cell lines

Cells were stably infected with shRNA against p38 (CCGGGTACTTCCTGTGTACTCTTTAC TCGAGTAAAGAGTACACAGGAAGTACTTTTTG), TRIM28-1 (CCGGGTACTGTCTATT GCAACGTCTCGAGACGTTGCAATAGACAGTACTTTTTG), TRIM28-2 (CCGGCTGAG GACTACAACCTTATCTCGAGATAAGGTTGTAGTCCTCAGTTTTTG) or scramble control in the pLKO.1 lentiviral background. For CRISPR–Cas9 system-mediated gene knockout, we used guide RNAs against p38 (CACCGCACAAAAACGGGGTTACGTG (F), AAACCCACACGTAACCCCGTTTTTGTGC (R)), MK2 (CACCGGAACTGCGGGAACTG CTGCG (F), AAACCGCAGCAGTTCCCGCAGTTCC (R)), TRIM28 (CACCGCACATCCC TGCTTCTCGAAG (F), AAACCTTCGAGAAGCAGGGATGTGC (R)) and MKK3 (CACCG ACAGGGTTCACTGCGCAGAT (F), AAACATCTGCGCAGTGAACCCTGTC (R)) in the Lenti-CRISPR v2 lentiviral background. TRIM28 KO HT-29 cells were generated by CRISPR technology and TRIM28 knock down FADD-deficient Jurkat cells were generated by shRNA. RIPK1^−/−^ and MLKL^−/−^ MEFs were generated by infecting MEFs with lentiviruses carrying Lenti-CRISPR v2-sgRIPK1 and Lenti-CRISPR v2-sgMLKL. For viral packaging, HEK293T cells were transfected with different vectors using Polyethylenimine (PEI, Polysciences, 23966-2). Viral supernatant fractions were collected at 48 h post-transfection. Cleared supernatant fraction with virus particles was filtered through a 0.45 μm filter. Polybrene (8 mg/mL) was supplemented to viral supernatant fractions at a final concentration of 8 μg/mL. Twenty-four hours after infection, cells stably expressing shRNA or sgRNA were obtained by selection with 5 μg/mL puromycin.

### Construction, transfection of plasmids, and reconstitution cell lines

Transient transfections of HEK293T cells were performed using PEI according to the manufacturer’s instructions. In brief, HEK293T cells were seeded in 6-well plates. When cells were 95% confluent, plasmids were transfected using PEI reagent at a ratio of 1:3, and each well was transfected with a total of 2 μg DNA per well for 24 h. Full-length cDNAs for human MLKL and TRIM28 were PCR-amplified from HT-29 cells and cloned into pcDNA3.1 vector using Q5® High-Fidelity DNA Polymerase (NEB, M0491) with appropriate tags. MLKL T357E/S358D and FKBP F36V were PCR-amplified from corresponding wild-type sequences with point mutation primers using PfuUltra II Fusion HS DNA polymerase (Agilent, 600674), then both cloned into pMSCV plasmid with Flag tag using ClonExpress II One Step Cloning Kit (Vazyme Biotech Co.,Ltd, C112) and transfected into HT-29 cells, and the reconstitution product was named as acMLKL. Tet-On vector carrying Flag-MLKL-Q356A was transfected individually into HEK293T cells, in which system MLKL oligomerization could be induced by doxycycline. For add-backs, TRIM28 WT, S473A, S473D and S473E sequences were cloned into pMSCV vector also using ClonExpress II One Step Cloning Kit. TRIM28 WT, S473A, S473D and S473E were reconstituted into TRIM28 KO HT-29 and TRIM28 knock down FADD-deficient Jurkat cells. Flag-p38 was reconstituted into p38 KO HT-29 cells. All plasmids were verified by DNA sequencing.

### Immunoblotting and co-immunoprecipitation (Co-IP)

Antibodies against the following proteins were used for western blot analysis: RIPK1 (Homemade), phospho-RIPK1 (S166) (CST, 65746), MLKL (Homemade; Abcam, ab183770; GeneTex, GTX107538), phospho-MLKL (T357/S358) (Abcam, ab187091), TRIM28 (Abcam, ab22553; CST, 4123), phospho-TRIM28 (S473) (Biolegend, 644602; Biolegend, 654102), phospho-TRIM28 (S824) (Abcam, ab70369), H2A.X (CST, 2595), phospho-H2A.X (S139) (Millipore, 05-636), Chk1(Santa Cruz, sc-8408), phospho-Chk1 (S296) (CST, 90178), phospho-Chk1 (S345) (CST, 2341), MK2 (CST, 3042), phospho-MK2 (T334) (CST,3007), p38 (CST, 9212), phospho-p38 (CST, 4511; CST, 9216), MKK3 (CST, 8535), MKK6 (CST, 8550), phospho-MKK3/MKK6 (CST, 12280), IKK (CST, 8943S), phospho-IKK (CST, 2078S), RIPK3 (Homemade), phospho-RIPK3 (CST, 93654), GFP (Santa Cruz, sc-9996), HA (SAB, T501), Actin (Santa Cruz, 81178), α-Tubulin (MBL, M175-3), GAPDH (Sigma-Aldrich, G5262), Goat anti-Mouse IgG (H + L) Cross-Adsorbed Secondary Antibody, Alexa Fluor 488 (Thermo Fisher Scientific, A11001), Goat anti-Rabbit IgG (H + L) Cross-Adsorbed Secondary Antibody, Alexa Fluor 594 (Thermo Fisher Scientific, A11012), Goat anti-Rabbit IgG (H + L) Secondary Antibody, HRP (Thermo Fisher Scientific, 31460), Goat anti-Mouse IgG (H + L) Secondary Antibody, HRP (Thermo Fisher Scientific, 31430). For Co-immunoprecipitation, cell lysates were prepared in following lysis buffer: 50 mM Tris-HCl (pH 7.2), 150 mM NaCl, 1 mM EDTA, 1 mM EGTA, 0.5% NP40, 10% glycerol supplemented with 0.5 mM PMSF, 20 mM N-ethylmaleimide (N-EM), 1× protease inhibitor cocktail (Bimake, B14001), 10 mM β-glycerol phosphate, 5 mM NaF, and 200 μM Na_3_VO_4_. Cells were lysed on ice for 1 h and centrifuged at 15,000 rpm for 15 min at 4 °C. The cell lysates were incubated with indicated antibody overnight at 4 °C and immunocomplex was captured by protein G agarose (Thermo Fisher Scientific, 20399). After extensive washes, beads were boiled in loading buffer and eluted products were separated by SDS-PAGE, which were transferred to nitrocellulose membrane and analyzed with indicated antibodies.

### Immunofluorescence

For immunofluorescence analysis, HT-29 cells were grown on coverslips. The cells were fixed with 4% paraformaldehyde, permeabilized with 0.1% TritonX-100 (Sigma-Aldrich, T8787) and blocked with 5% bovine serum albumin (BSA). Blocked cells were incubated with indicated primary antibody overnight at 4 °C. Cells were then incubated with secondary antibody for 1 h at room temperature. Fluorescence imaging was done on Leica SP8 Confocal System.

### Quantitative reverse-transcription PCR

Total RNA was extracted with TRIzol Reagent (Thermo Fisher Scientific, 15596026) and reverse transcription was performed with Reverse Transcriptase M-MLV (RNase H-) (Takara, H2640A). Real-time PCR primer sequences were as follows (5’−3’): IL8: CAGTTTTGCCAAGGAGTGCT (F), ACTTCTCCACAACCCTCTGC (R); TNFα: CCCAGGCAGTCAGATCATCT (F), GGACCTGGGAGTAGATGAGG (R); Actin: GGCACCACACCTTCTACAATGAGC (F), CCTGGATGGCTACGTACATGGCTG (R). Quantitative PCR was performed on Quant Studio 6 Flex Real-Time PCR System (Applied Biosystems) using 2×SYBR Green Master Mix reagent (Bimake, B21202). *Actin* was used as a reference gene. Data were analyzed according to the 2^–ΔΔCT^ method.

### Analysis of cytotoxicity and viability

The rates of cell death were measured in triplicate in a 384-well plate using ToxiLight Non-destructive Cytotoxicity BioAssay Kit (Lonza, LT07-217). General cell survival was measured by the ATP luminescence assay CellTiterGlo (Promega, G7573) according to the manufacturer’s protocol and the results were expressed as percentages of luminescence intensity per well after comparing to that of the viability in the untreated wells. The intensity of luminescence was determined in an EnSpire Multimode Plate Reader (PerkinElmer). Data were collected using PerkinElmer EnVision Manager Version 1.13 software.

### RNA-Seq

One microgram of total RNA was used for stranded mRNA library preparation according to the manufacturer’s protocol (http://www.interchim.fr/ft/B/B0EJM0.pdf). Libraries were quantified using both Qubit and Agilent 2100 Bioanalyzer (Agilent RNA 6000 Nano Kit) to detect the total RNA samples concentration. Libraries were run on NextSeq at 150 nucleotide and pair-end sequence read length. Reads were mapped to the Human Genome GRCh38.p12 with STAR using the default parameters. Reads were assigned to all gene in the GENCODE version 29 GTF file using the featureCounts. Reads per gene were then analyzed using the DESeq2 R package from Bioconductor. Genes upregulated and downregulated with TSZ treated were selected by comparing DMSO-treated T28 cells cultivated at 37 °C. Expression differences were determined using drug as the independent categorical variable modeled with DESeq2 using the DESeq analysis for statistical significance. Differentially expressed genes were selected using the absolute log2 fold change of at least 2 as calculated by the DESeq2 package and a Benjamini–Hochberg adjusted *p*-value of <0.05. The lists of differentially expressed genes were used for DAVID GO analysis, comparing to the background list of all genes.

### Mass spectrometry and data analysis

The SILAC strategy was used for quantification of RIPK1 kinase-dependent phosphoproteome. The “light (K0, R0)”, “medium (K4, R6)” and “heavy (K8, R10)” labeled cells were treated with DMSO, TNFα and TNFα + Nec-1s, respectively. Cells were harvested in lysis buffer after treatments for 15 min, 0.5, 2, and 4 h. The protein concentrations were measured. Equal amount of proteins in three differently labeled cells were mixed for each time point and trypsin digested. The resulted peptides were separated by high-pH reversed-phase fractionation. A part of peptides of each fractionation were directly analyzed by mass spectrometry for total proteome analysis, and the other part of peptides were subjected to phosphopeptide enrichment by TiO_2_ and analyzed by mass spectrometry for phosphoproteome analysis. The peptides were analyzed on Easy-nanoLC 1000-Orbitrap Fusion and Q Exactive HF-X Hybrid Quadrupole-Orbitrap Mass Spectrometer (Thermo Scientific). Mass spectrometry was based on Data Dependent Acquisition (DDA). Protein and phosphosites identification and quantification were performed by MaxQuant [35]. The mass spectra were searched against the Swissprot human protein database (released in Jun 2018). The precursor and fragment mass tolerances were set as 20 ppm. The unique peptides plus razor peptides were included for quantification. The cysteine carbamidomethylation was set as a static modification, and the N-acetylation, S/T/Y phosphorylation and methionine oxidation were set as variable modifications. The false discovery rate (FDR) at peptide spectrum match levels was based on target-decoy and controlled below 1%. “Match between runs” were used during data analysis. In phosphoproteome analysis, unpaired two-tailed Student’s *t*-test was applied in comparing significant difference between experimental group and control group. Data consolidation, curve fitting, clustering analysis, frequency analysis, heat map plotting and statistical analysis were performed using R, Gitools, Perseus [36], OriginPro, Graphpad or Microsoft Excel 2013 software. Gene functional clustering analysis was performed using David (https://david.ncifcrf.gov/home.jsp), Metascape (https://metascape.org/gp/index.html#/main/step1), cytoscape [37] and Gene Analytics^TM^ (https://geneanalytics.genecards.org/).

### Statistical analysis

The cell data are presented as mean ± SEM (standard error of mean) of triplicate wells from one representative experiment. All immunoblots from cell samples were repeated at least three times independently with similar results. All cell viability data and quantitative PCR data were analyzed with unpaired two-tailed Student’s *t*-test. Differences were considered statistically significant if *p* < 0.05. *p < 0.05, **p < 0.01, or ***p < 0.001; NS, not significant.

## Supplementary information

supplementary figure legends

supplementary figures

Supplementary tables

## Data Availability

The mass spectrometry proteomics data have been deposited to the ProteomeXchange Consortium (http://proteomecentral.proteomexchange.org) via the iProX partner repository [38] with the dataset identifier PXD025313. The RNAseq data have been deposited to Gene Expression Omnibus (https://www.ncbi.nlm.nih.gov/geo/) with the dataset identifier GSE171214.

## References

[1] Zhu K, Liang W, Ma Z, Xu D, Cao S, Lu X (2018). Necroptosis promotes cell-autonomous activation of proinflammatory cytokine gene expression. Cell Death Dis.

[2] Shan B, Pan H, Najafov A, Yuan J (2018). Necroptosis in development and diseases. Genes Dev..

[3] Degterev A, Huang Z, Boyce M, Li Y, Jagtap P, Mizushima N (2005). Chemical inhibitor of nonapoptotic cell death with therapeutic potential for ischemic brain injury. Nat Chem Biol..

[4] Degterev A, Hitomi J, Germscheid M, Ch’en IL, Korkina O, Teng X (2008). Identification of RIP1 kinase as a specific cellular target of necrostatins. Nat Chem Biol..

[5] Mifflin L, Ofengeim D, Yuan J (2020). Receptor-interacting protein kinase 1 (RIPK1) as a therapeutic target. Nat Rev Drug Discov.

[6] Ito Y, Ofengeim D, Najafov A, Das S, Saberi S, Li Y (2016). RIPK1 mediates axonal degeneration by promoting inflammation and necroptosis in ALS. Science.

[7] Ofengeim D, Mazzitelli S, Ito Y, DeWitt JP, Mifflin L, Zou C (2017). RIPK1 mediates a disease-associated microglial response in Alzheimer’s disease. Proc Natl Acad Sci USA.

[8] Koper MJ, Van Schoor E, Ospitalieri S, Vandenberghe R, Vandenbulcke M, von Arnim CAF (2019). Necrosome complex detected in granulovacuolar degeneration is associated with neuronal loss in Alzheimer’s disease. Acta Neuropathol.

[9] Degterev A, Maki JL, Yuan J (2013). Activity and specificity of necrostatin-1, small-molecule inhibitor of RIP1 kinase. Cell Death Differ.

[10] Degterev A, Ofengeim D, Yuan J (2019). Targeting RIPK1 for the treatment of human diseases. Proc Natl Acad Sci USA.

[11] Yuan J, Amin P, Ofengeim D (2019). Necroptosis and RIPK1-mediated neuroinflammation in CNS diseases. Nat Rev Neurosci..

[12] Newton K, Dugger DL, Maltzman A, Greve JM, Hedehus M, Martin-McNulty B (2016). RIPK3 deficiency or catalytically inactive RIPK1 provides greater benefit than MLKL deficiency in mouse models of inflammation and tissue injury. Cell Death Differ.

[13] Ofengeim D, Yuan J (2013). Regulation of RIP1 kinase signalling at the crossroads of inflammation and cell death. Nat Rev Mol Cell Biol..

[14] Chen G, Goeddel DV (2002). TNF-R1 signaling: a beautiful pathway. Science..

[15] Amin P, Florez M, Najafov A, Pan H, Geng J, Ofengeim D (2018). Regulation of a distinct activated RIPK1 intermediate bridging complex I and complex II in TNFalpha-mediated apoptosis. Proc Natl Acad Sci USA.

[16] He S, Wang L, Miao L, Wang T, Du F, Zhao L (2009). Receptor interacting protein kinase-3 determines cellular necrotic response to TNF-alpha. Cell..

[17] Zhang DW, Shao J, Lin J, Zhang N, Lu BJ, Lin SC (2009). RIP3, an energy metabolism regulator that switches TNF-induced cell death from apoptosis to necrosis. Science..

[18] Sun L, Wang H, Wang Z, He S, Chen S, Liao D (2012). Mixed lineage kinase domain-like protein mediates necrosis signaling downstream of RIP3 kinase. Cell..

[19] Iyengar S, Farnham PJ (2011). KAP1 protein: an enigmatic master regulator of the genome. J Biol Chem.

[20] Bolderson E, Savage KI, Mahen R, Pisupati V, Graham ME, Richard DJ (2012). Kruppel-associated Box (KRAB)-associated co-repressor (KAP-1) Ser-473 phosphorylation regulates heterochromatin protein 1beta (HP1-beta) mobilization and DNA repair in heterochromatin. J Biol Chem.

[21] Wang Y, Li J, Huang Y, Dai X, Liu Y, Liu Z (2017). Tripartite motif-containing 28 bridges endothelial inflammation and angiogenic activity by retaining expression of TNFR-1 and -2 and VEGFR2 in endothelial cells. FASEB J: Off Publ Federation Am Societies Exp Biol.

[22] Morrison DK (2012). MAP kinase pathways. Cold Spring Harbor Perspect. Biol.

[23] Cuadrado A, Nebreda AR (2010). Mechanisms and functions of p38 MAPK signalling. Biochemical J.

[24] Ofengeim D, Ito Y, Najafov A, Zhang Y, Shan B, DeWitt JP (2015). Activation of necroptosis in multiple sclerosis. Cell Rep.

[25] Krishnan RK, Nolte H, Sun T, Kaur H, Sreenivasan K, Looso M (2015). Quantitative analysis of the TNF-alpha-induced phosphoproteome reveals AEG-1/MTDH/LYRIC as an IKKbeta substrate. Nat Commun.

[26] Hu C, Zhang S, Gao X, Gao X, Xu X, Lv Y (2012). Roles of Kruppel-associated box (KRAB)-associated co-repressor KAP1 Ser-473 phosphorylation in DNA damage response. J Biol Chem.

[27] Yoon S, Bogdanov K, Kovalenko A, Wallach D (2016). Necroptosis is preceded by nuclear translocation of the signaling proteins that induce it. Cell Death Differ.

[28] Friedman JR, Fredericks WJ, Jensen DE, Speicher DW, Huang XP, Neilson EG (1996). KAP-1, a novel corepressor for the highly conserved KRAB repression domain. Genes Dev.

[29] Zhong CQ, Li Y, Yang D, Zhang N, Xu X, Wu Y (2014). Quantitative phosphoproteomic analysis of RIP3-dependent protein phosphorylation in the course of TNF-induced necroptosis. Proteomics.

[30] Trempolec N, Dave-Coll N, Nebreda AR (2013). SnapShot: p38 MAPK substrates. Cell.

[31] Li J, McQuade T, Siemer AB, Napetschnig J, Moriwaki K, Hsiao YS (2012). The RIP1/RIP3 necrosome forms a functional amyloid signaling complex required for programmed necrosis. Cell.

[32] Fisk M, Gajendragadkar PR, Maki-Petaja KM, Wilkinson IB, Cheriyan J (2014). Therapeutic potential of p38 MAP kinase inhibition in the management of cardiovascular disease. Am J Cardiovasc Drugs.

[33] Aashaq S, Batool A, Andrabi KI (2019). TAK1 mediates convergence of cellular signals for death and survival. Apoptosis: Int J Program Cell Death.

[34] Geng J, Ito Y, Shi L, Amin P, Chu J, Ouchida AT (2017). Regulation of RIPK1 activation by TAK1-mediated phosphorylation dictates apoptosis and necroptosis. Nat Commun.

[35] Tyanova S, Temu T, Cox J (2016). The MaxQuant computational platform for mass spectrometry-based shotgun proteomics. Nat Protoc.

[36] Tyanova S, Temu T, Sinitcyn P, Carlson A, Hein MY, Geiger T (2016). The Perseus computational platform for comprehensive analysis of (prote)omics data. Nat Methods.

[37] Shannon P, Markiel A, Ozier O, Baliga NS, Wang JT, Ramage D (2003). Cytoscape: a software environment for integrated models of biomolecular interaction networks. Genome Res.

[38] Ma J, Chen T, Wu S, Yang C, Bai M, Shu K (2019). iProX: an integrated proteome resource. Nucleic Acids Res.

