# Supplementary material for: Quantitative analysis of phosphoproteome in necroptosis reveals a role of TRIM28 phosphorylation in promoting necroptosis-induced cytokine production
Source: Cell Death Dis. 2021 Oct 23;12(11):994. doi: 10.1038/s41419-021-04290-7 (PMC8542044; doi:10.1038/s41419-021-04290-7)
Supplement: Supplementary file 1 — supplementary figure legends [file 41419_2021_4290_MOESM1_ESM.docx]

**Supplementary Figure legends**

**Sup Figure 1**

(A) A time course analysis of cell viability of FADD-deficient Jurkat cells treated with TNFα in the presence or absence of Nec-1s by CellTiter-Glo. Data were presented as mean±SEM.

(B) FADD-deficient Jurkat cells were treated with TNFα in the presence or absence of Nec-1s. The lysates were immunoprecipitated by RIPK1 antibody and analyzed by western blotting using indicated antibodies.

(C) The SILAC strategy for quantification of necroptosis phosphoproteome. The “light”, “medium” and “heavy” labeled cells were treated with vehicle only, TNFα and TNFα+ Nec-1s, respectively. Cells were harvested in lysis buffer after treatments for 15min, 0.5h, 2h and 4h. The protein concentrations were measured. Equal amount of proteins in three differently labeled cells were mixed for each time point and trypsin digested. The resulted peptides were separated by high pH reversed-phase fractionation. A part of peptides of each fractionation were directly analyzed by mass spectrometry for total proteome analysis, and the other part of peptides were subjected to phosphopeptide enrichment by TiO_2_ and analyzed by mass spectrometry for phosphoproteome analysis.

(D) Coefficient of variations (CVs) among 6 replicates of phosphosite quantifications in all the 4 time points of TNFα treatments in the presence or absence of Nec-1s.

(E) Frequency curves of observed peptide and protein ratios used for phosphosites (blue) and proteome (orange) quantification.

TNFα, 20ng/mL; Nec-1s, 10μM.

**Sup Figure 2**

(A) Volcano plots of statistical significance against fold changes of phosphosites in temporal induction of TNFα. We defined the phosphosites with log_2_Ratios ≥0.485 or ≤-0.485 and *p-*value<0.05 as significantly upregulated (orange dots) or downregulated (green dots) by TNFα stimulation, respectively.

(B) The numbers of phosphosites up and down-regulated by TNFα stimulation in the presence or absence of Nec-1s.

(C) The percentage of phosphosites inhibited or rescued by treatment with Nec-1s among TNFα up-regulated or down-regulated phosphosites, respectively.

TNFα, 20ng/mL; Nec-1s, 10μM.

**Sup Figure 3**

(A-D) HT-29 cells were treated with indicated compounds at indicated time course. The levels of specified proteins were analyzed by western blotting using indicated antibodies.

TNFα, 20ng/mL; zVAD, 25μM; SM164, 50nM; Nec-1s, 10μM; CHX, 1ng/mL.

**Sup Figure 4**

(A) HT-29 cells were treated with Etoposide alone or plus Nec-1s, GSK’872 and NSA for 4h and 8h, respectively. The specified protein levels were analyzed by western blotting using indicated antibodies. *, nonspecific bands.

(B) HT-29 cells were treated with TSZ or TSZ+Nec-1s in a time course. The cell lysates were analyzed by western blotting using phospho-H2A.X and basal H2A.X antibodies.

(C-D) FADD-deficient Jurkat cells (C) and HT-29 cells (D) were treated with indicated compounds for 4h. The specified protein levels were analyzed by western blotting using indicated antibodies.

(E) Domain structures of human MLKL protein and deletion constructs used to investigate the binding specificity of individual domains of MLKL. FL, full-length MLKL, 1-471; 4HB, N-terminal four-helix bundle (4HB) 'executioner' domain, 1-125; CC, coiled-coil region, 126-178; PKD, pseudo-kinase domain, 179-471.

(F) Immunoblot of HEK293T cells captured with antibody to FLAG after transfection of Flag-TRIM28 and Myc-MLKL deletion mutants.

(G) Immunoblot of HEK293T cells captured with antibody to Myc after transfection of Flag-TRIM28 and Myc-MLKL deletion mutants. Cell lysates were captured with magnetic beads conjugated with antibody to Flag or Myc for immunoprecipitation, and the resulting beads were analyzed by immunoblot.

TNFα, 20ng/mL; zVAD, 25μM; SM164, 50nM; Nec-1s, 10μM; GSK’872, 10μM; NSA, 2μM; Etoposide, 20μM; Chk1 inhibitor MK-8776, 2μM; Chk1 inhibitor PD-407824, 2μM.

**Sup Figure 5**

(A) HT-29 cells stably expressing shSTK4, shWNK, shPRKDC, shADK, shPRKCI were treated with TSZ for 6h. The specified protein levels were analyzed by western blotting using indicated antibodies. The expression levels of mRNA was determined by RT-PCR.

(B) HT-29 cells were transfected with an expression vector of flag-tagged MLKL fused with two AP20187-binding (FKBPv) domains. RIPK3 was knocked down by siRNA in flag-FKBP-MLKL-HT-29 cells. Wild-type and RIPK3-kd cells were pretreated with Nec-1s for 1h followed by treatment with AP20187 to induce MLKL oligomerization. Cell lysates were separated by non-reducing SDS/PAGE and analyzed by western blotting using MLKL antibody. Other proteins were separated by reducing SDS/PAGE and analyzed by western blotting using indicated antibodies. acMLKL, oligomerizable MLKL; endo, endogenous.

(C) HT-29 cells were treated with TSZ in the presence or absence of 5Z-7 and Nec-1s at indicated time course. The specified protein levels were analyzed by western blotting using indicated antibodies.

(D-E) MKK3 KO (D) and MK2 KO (E) HT-29 cells were treated with TSZ or TSZ+Nec-1s at indicated time course. The specified protein levels were analyzed by western blotting using indicated antibodies.

(F) MKK3 and MKK6 were knocked down by siRNA in HT-29 cells and cells were treated with TSZ for 2h and 6h. The cell lysates were analyzed by western blotting using indicated antibodies.

TNFα, 20ng/mL; zVAD, 25μM; SM164, 50nM; Nec-1s, 10μM; 5Z-7, 500nM; AP20187, 100nM.

**Sup Figure 6**

(A) TRIM28 was knocked out in HT-29 cells using CRISPR technology and the expression of TRIM28 in the TRIM28 KO HT-29 cells was complemented using expression vectors for TRIM28 WT, S473A, S473D and S473E. The knockout efficiency and reconstituted expression levels were determined by western blotting.

(B) TRIM28 was knocked down in FADD-deficient Jurkat cells by shRNA and the expression of TRIM28 in the TRIM28 knockdown cells was complemented using expression vectors for TRIM28 WT, S473A, S473D and S473E. The knockdown efficiency and reconstituted expression levels were determined by western blotting.

(C-D) WT, TRIM28 KO and TRIM28 WT, S473A, S473D and S473E reconstituted HT-29 cells were treated with TSZ for 12h in the presence or absence of Nec-1s. The cell death was measured by ToxiLight assay (C). The cell viability was determined by CellTiter-Glo (D).

(E-G) WT, TRIM28 knockdown and TRIM28 WT, S473A, S473D and S473E reconstituted FADD-deficient Jurkat cells were treated with TNFα (E) or TS (F) or TC (G) for indicated time points. The cell viability was measured by CellTiter-Glo.

(H) FADD-deficient Jurkat cells stably expressing shTRIM28 were treated with TNFα for indicated time points in the presence or absence of Nec-1s. The cell lysates were separated by non-reducing SDS/PAGE and the protein levels of MLKL was analyzed by western blotting.

(I) WT, TRIM28 knockdown and TRIM28 WT, S473A, S473D and S473E reconstituted FADD-deficient Jurkat cells were treated with TNFα for 10h. The protein expression levels were detected by western blotting using indicated antibodies.

(J) WT, TRIM28 KO and TRIM28 WT, S473A, S473D and S473E reconstituted HT-29 cells were treated with TSZ for 12h in the presence or absence of Nec-1s. The cell lysates were separated by non-reducing SDS/PAGE and the protein levels were analyzed by western blotting using indicated antibodies.

Error bar, s.e.m. **, *t*-test *p*<0.01, n=3, n.s., not significant. TNFα, 20ng/mL; zVAD, 25μM; SM164, 50nM; Nec-1s, 10μM; CHX, 1ng/mL.

**Sup Figure 7**.

(A) Volcano plots of statistical significance against fold changes of the genes upon induction of necroptosis by treatment with TSZ in TRIM28 WT complemented TRIM28 KO HT-29 cells. We display genes statistically significant (*p*<0.05) and log_2_Ratios >=2 in yellow dots, while <=-2 in blue dots. Statistical analysis was done by *t*-test, n=3.

(B) A pathway analysis of upregulated genes. The top10 significant pathways were plotted against –log_10_ (*P-*value).
