# Supplementary material for: Quantitative analysis of phosphoproteome in necroptosis reveals a role of TRIM28 phosphorylation in promoting necroptosis-induced cytokine production
Source: Cell Death Dis. 2021 Oct 23;12(11):994. doi: 10.1038/s41419-021-04290-7 (PMC8542044; doi:10.1038/s41419-021-04290-7)

Supplementary Figure 1

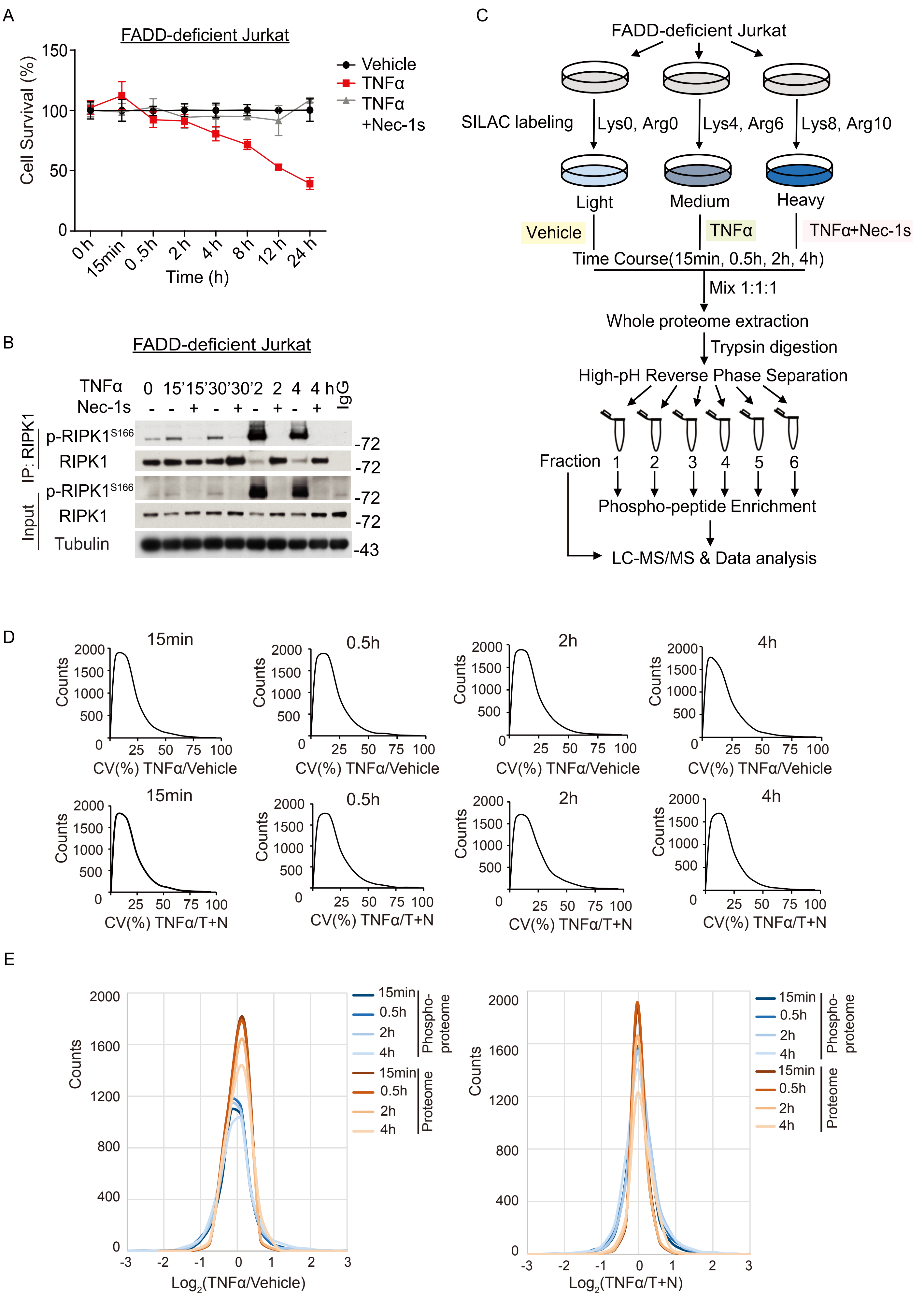

Supplementary Figure 2

A

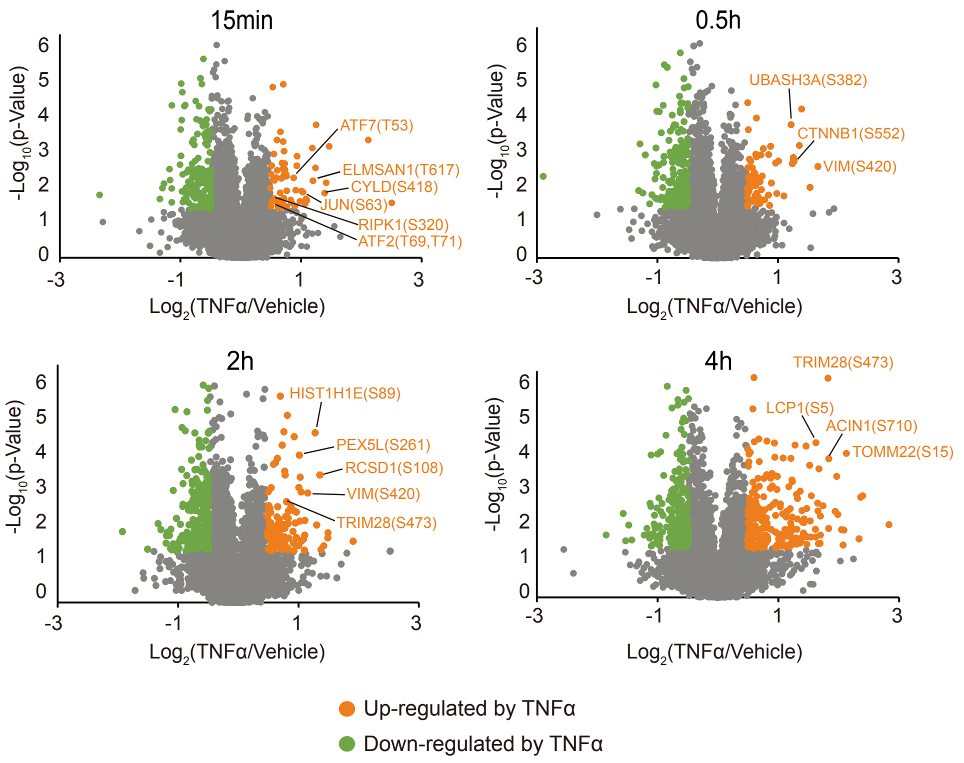

B

Time points	Number of Phospho-sites			
	Upregulated by TNFα	Inhibited by Nec-1s	Downregulated by TNFα	Rescued by Nec-1s
15min	67	28	177	33
0.5h	76	33	201	40
2h	136	95	252	71
4h	236	194	255	105

C

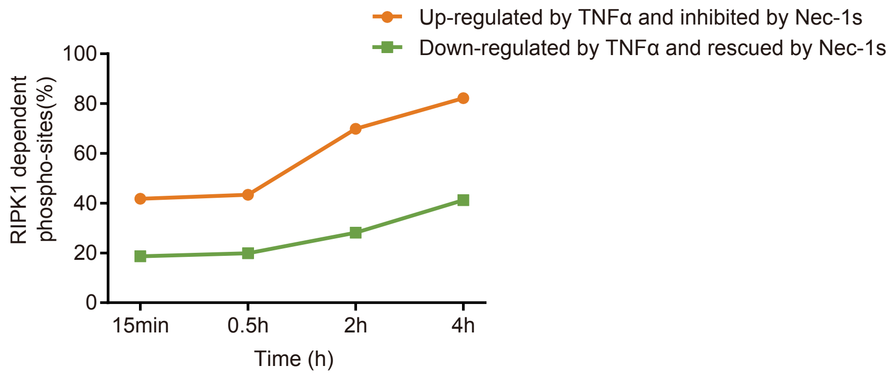

Supplementary Figure 3

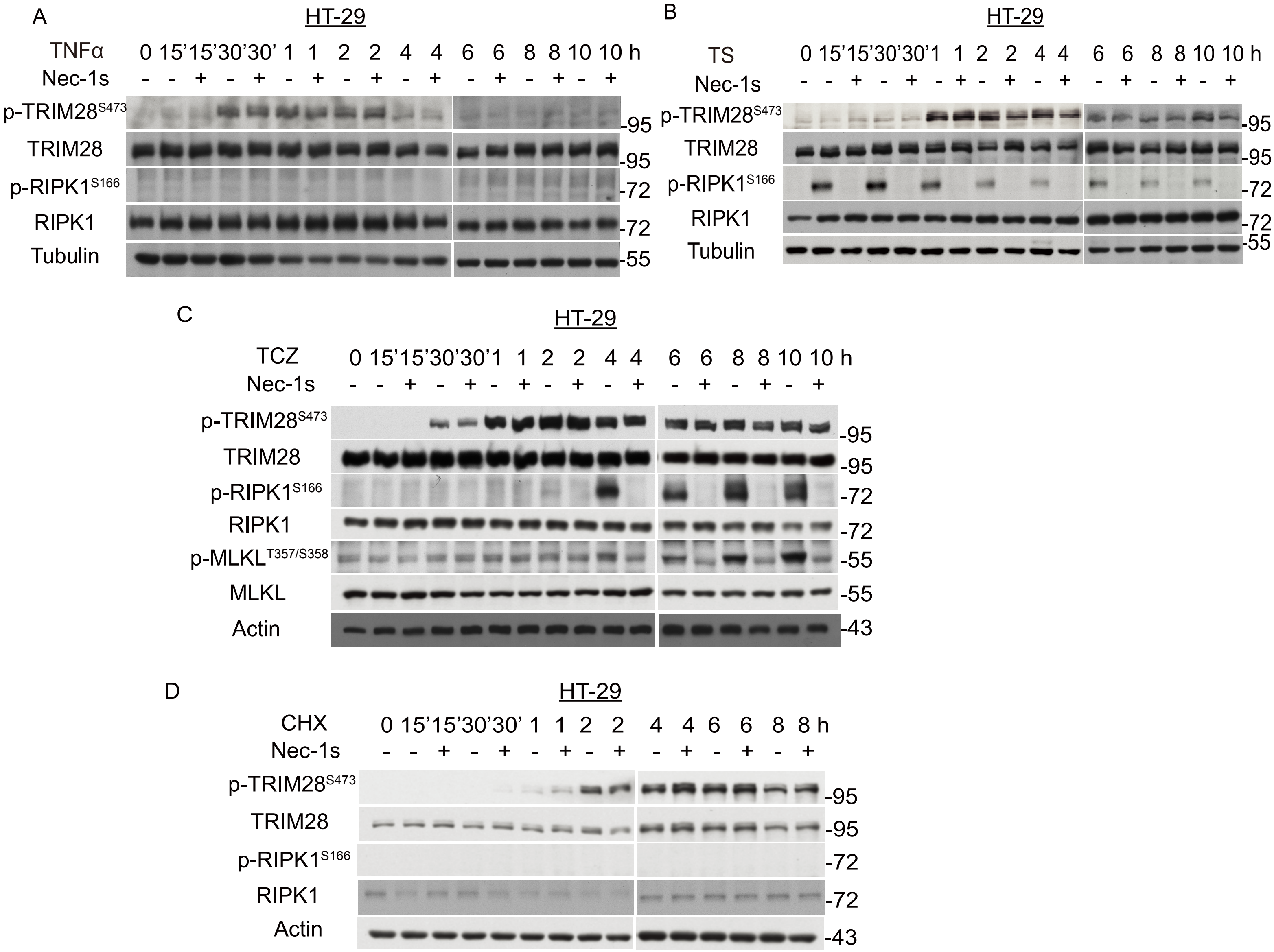

Supplementary figure 4

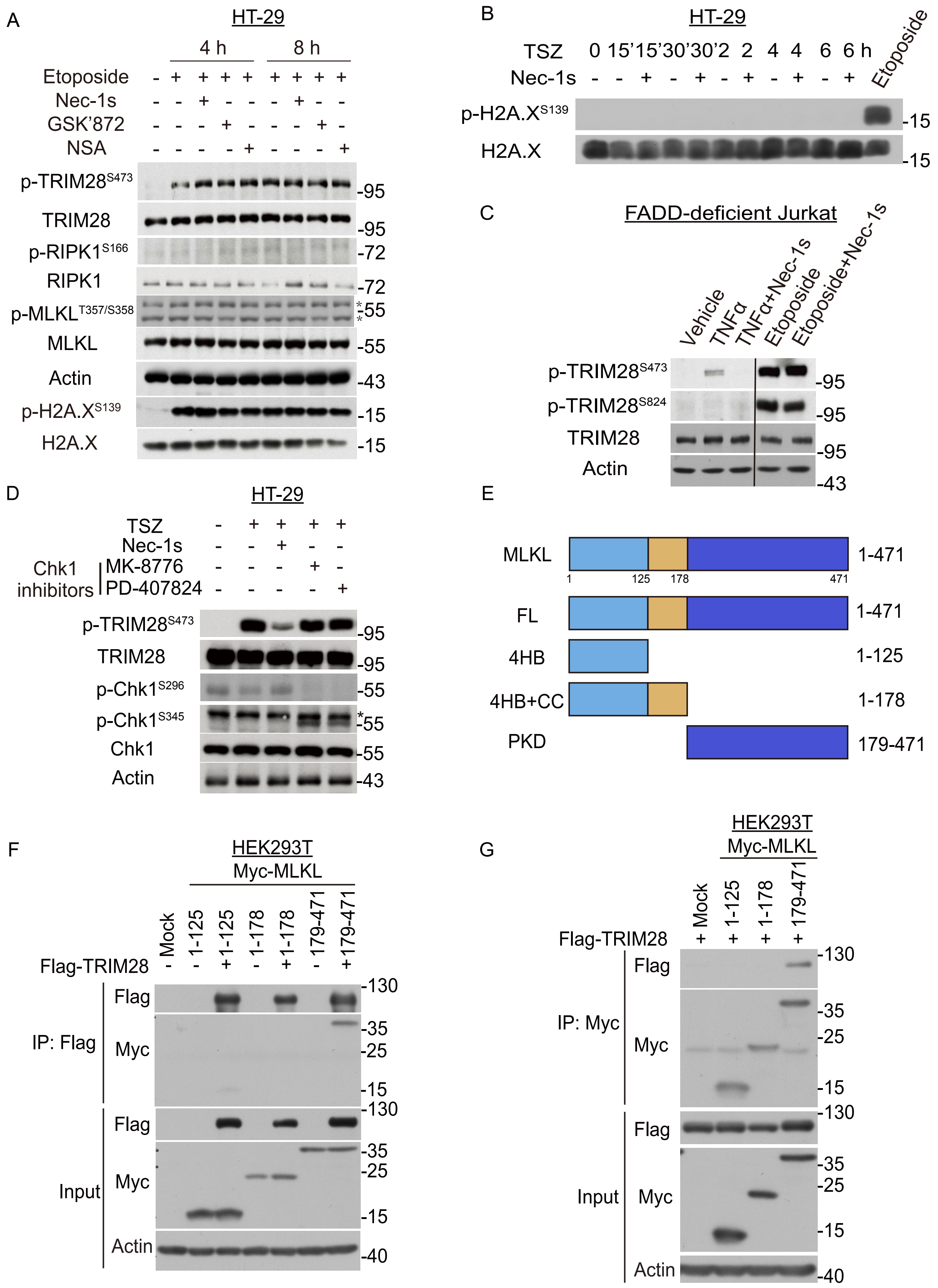

Supplymentary figure 5

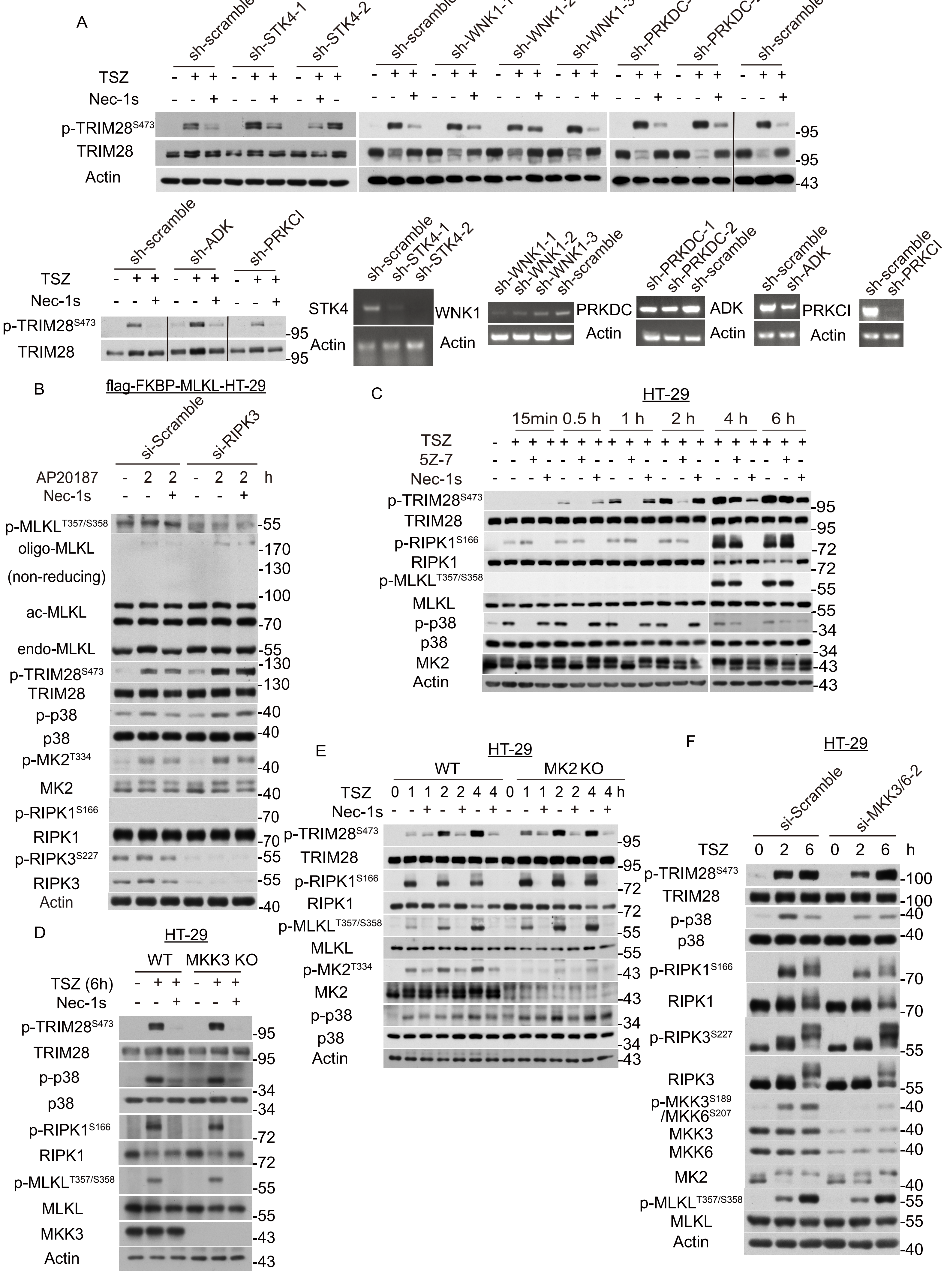

Supplementary Figure 6

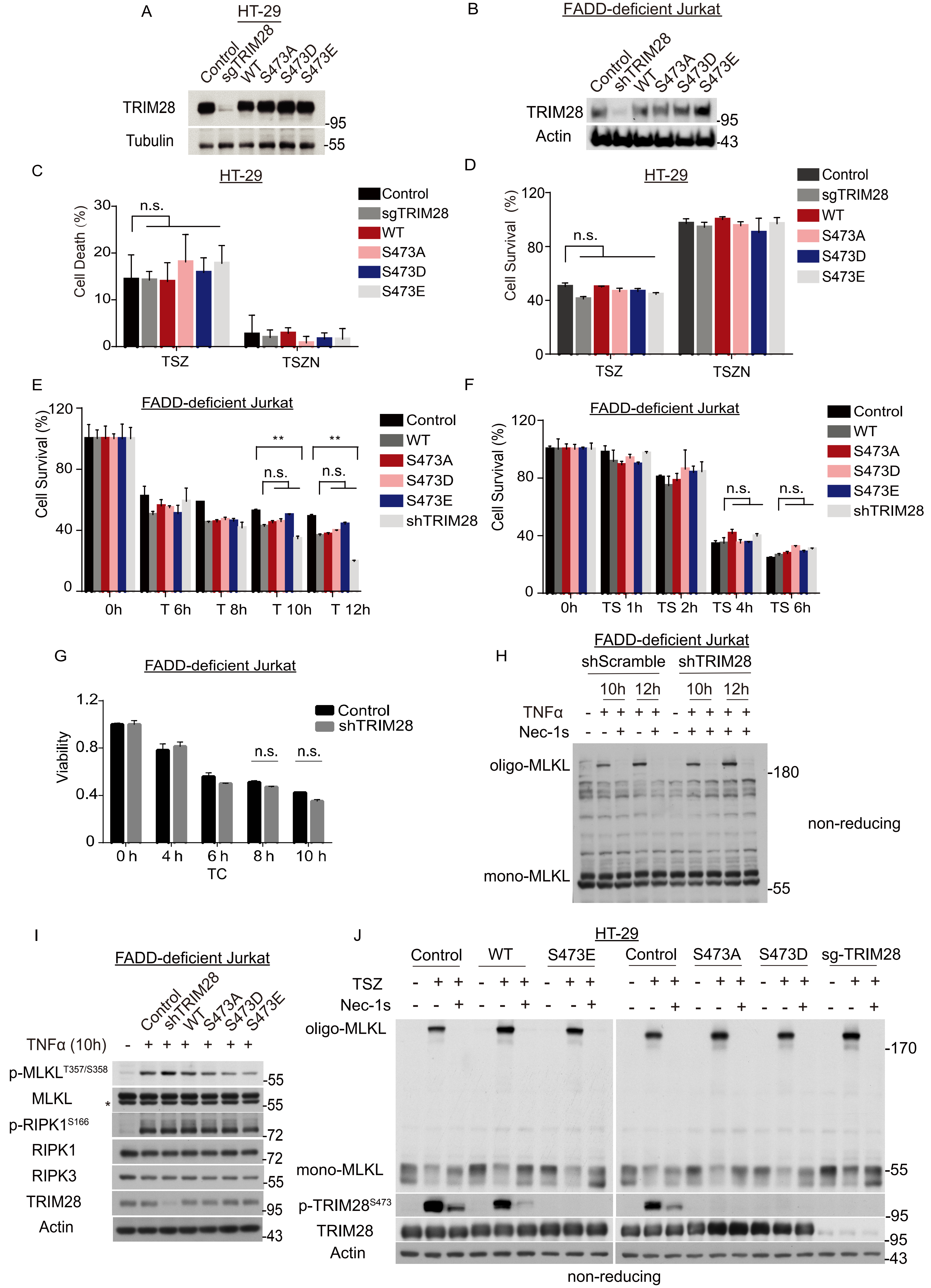

Supplementary Figure 7

A

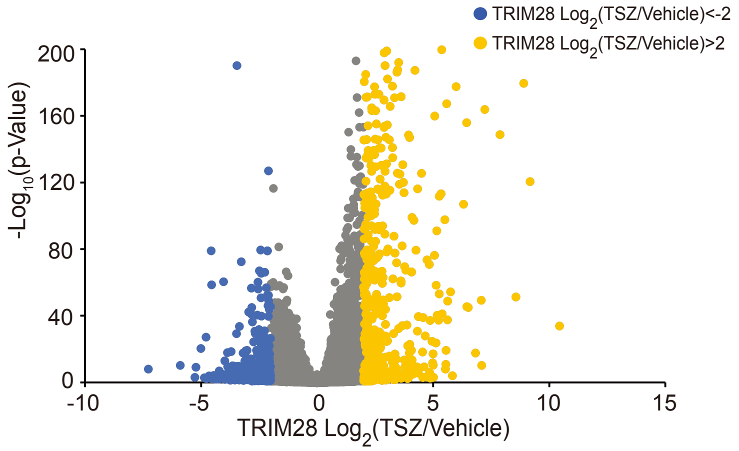

B

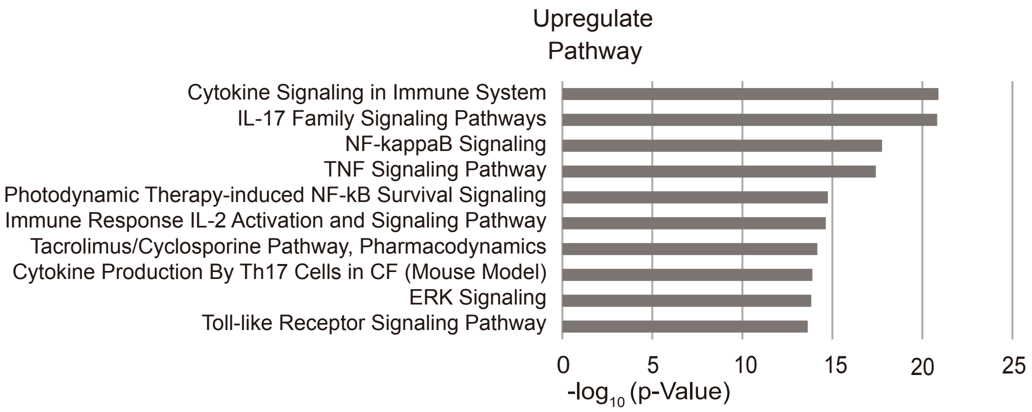

Supplement: Supplementary file 2 — supplementary figures [file 41419_2021_4290_MOESM2_ESM.pdf]
